# Supplementary material for: A Metagenomic Investigation of the Viruses Associated with Shiraz Disease in Australia
Source: Viruses. 2023 Mar 17;15(3):774. doi: 10.3390/v15030774 (PMC10056481; doi:10.3390/v15030774)
Supplement: Supplementary file 1 [file viruses-15-00774-s001.zip › viruses-2265361-supplementary.pdf]

Supplementary Material

Table S1. Sampling time, numbers of canes or petioles sampled per grapevine, tissue type and sample codes for samples used in the reverse transcription polymerase chain reaction (RT-PCR) and metagenomics high throughput sequencing (Meta-HTS) experiments. Samples were collected from four vineyards between May 2018 and December 2020.

Sampling time	Vineyard locations[3]	tissue type	No. of canes/petioles per grapevine	Total no. of samples	Sample code
May to July 2018[1]	WIL, LC and BV	autumn or dormant canes	3	66	WIL1-30, LC1-30 and BV1-6
March 2019[1,2]	WIL, LC and BV	petioles	5	66	WIL1-30, LC1-30 and BV1-6
August 2020[1,2]	WIL	dormant canes	5	50	WIL31-81 (exclude WIL46)
December 2020[1,2]	WIL and CV	petioles	5	9	Cabw1, 2, 11 and 12, Merlot1 (WIL), Shiraz_OR_P5 and P6, CabSA125_R3V30 and R3V44 (CV)

[1] Samples for RT-PCR virus detection.
[2] Samples for metagenomics high throughput sequencing.
[3] WIL = Willunga, LC = Langhorne Creek, BV = Barossa Valley, CV= Coombe's Vineyard. All grapevines listed are var. Shiraz except Cabw = Cabernet Sauvignon clone SA125 from Willunga and CabSA125 = Cabernet Sauvignon clone SA125 from Coombe's Vineyard.

Table S2. Primers sequences for virus detection using endpoint RT-PCR.

Virus	Primers	Primer sequence (5′- 3′)	Annealing Temperature (°C)	Gene	Amplicon size	Reference
GVA	Ah587<br>Ac995	GACAAATGGCACACTACG<br>AAGCCTGACCTAGTCATCTTGG	56	CP	430bp	[52]
GVA	H7038<br>C7273	AGGTCCACGTTTGCTAAG<br>CATCGTCTGAGGTTTCTACTAT	56	RNA binding	273bp	[33]

GVA	GVAgroupIIIF[2]<br>GVAgroupIIIR[2]	GGAGAGGTAGATATAGTAGGACC<br>CTTCTTGCAGAGTCAAGGTC	56	CP	315	Modified from primer pair 6591F/6906R [33]
GLRaV-1	p35LR17589f<br>p35LR17763r	AATCCTATGCGTCAGTATGC<br>TGGCATCGTTGCTAAATTGAG	56	CP	174 bp	[53]
GLRaV-2	LR2-U2<br>LR2-L2	ATAATTCGGCGTACATCCCCACTT<br>GCCCTCCGCGCAACTAATGACAG	56	HSP 70-like protein	332 bp	[54]
GLRaV-3	HSP70<br>HSP7070	GGGTCAAGTGCTCTAGTTAAGGTCA<br>AAAGTGTCCACCAGTCTCAGTCC	58	HSP 70-like protein	167bp	[44]
GLRaV-3	LR3P-H420<br>LR3P-C629	GATTTAAGCGCGTTTTTCAGGAC<br>CGGCACGATCGTACTTTCTAA	58	Hsp90-like protein	210 bp	[55]
GLRaV-4 strain 4	LR4CP298F<br>LR4CP298R	CCTGTTACGCCGCCTACTAC<br>GGTGCTTGTCACTCTCCGAA	56	CP	298bp	This study
GLRaV-4 strain 5[1]	LR5-1F<br>LR5-1R	CCCGTGATACAAGGTAGGACA<br>CAGACTTCACCTCCTGTTAC	55	3′-URT	690bp	[56]
GLRaV-4 strain 6	LR6_CPF<br>LR6_CPR	AAATTCCGCGCCTMTTCAATG<br>ATGTCTGGGGCCACCTTAGT	58	CP	563bp	This study
GLRaV-4 strain 9	LR9F<br>LR9R	ACA GTG GTC GGC ATA AGA AAA G<br>ACACAA ACA TGC AGG CCA AAG	56	Hsp90-like protein	250bp	[57]
GRVFV	GRVFV237F<br>GRVFV237R	ACTGAGCTACAAGGTGAATTGC<br>AGCAACCCACTGGAAGGGGATGG	60	Polyprotein	237bp	[58]
GRSPaV	RSP48<br>RSP49	AGCTGGGATTATAAGGGAGGT<br>CCAGCCGTTCCACCACTAAT	56	CP	330bp	[59]
GVB	H6980<br>C7439	GTGCTAAGAACGTCTTCACAGC<br>ATCAGCAAACACGCTTGAACCG	56	RNA binding	460bp	[60]
RubiscoL internal control	RBCL-H535<br>RBCL-C705	CTTTCCAAGGCCCGCCTCA<br>CATCATCTTTGGTAAAATCAAGTCCA	56	N/A	171bp	[23]

[1] For Sanger sequencing only.
[2] Grapevine virus A phylogroup III specific RT-PCR assay.

Table S3. Symptom observation and the viruses detected in Shiraz grapevines at the Willunga, Langhorne Creek, Barossa, and Adelaide University (Coombe's) vineyards using endpoint RT-PCR.

Location	Symptoms	Variety and Clone	Virus detected by RT-PCR#	Number of grapevines with given virus [illegible]
Willunga	Asymptomatic	Shiraz BVRC12	GRSPaV	1
			GRVFV, GRSPaV	3
			GLRaV-4/6, GRSPaV	1
			GLRaV-4/9, GRSPaV	2
			GLRaV-4/6, GRVFV, GRSPaV	10
			GLRaV-4/9, GRVFV, GRSPaV	7
			GLRaV-4/6, GLRaV-4/9, GRVFV, GRSPaV	5
	LRD	Shiraz BVRC12	GLRaV-3, GRSPaV	1
			GLRaV-3, GLRaV-4/6, GRSPaV	1
			GLRaV-3, GRVFV, GRSPaV	7
			GLRaV-3, GLRaV-4/9, GRVFV, GRSPaV	10
			GLRaV-3, GLRaV-4/6, GRVFV, GRSPaV	4
			GLRaV-3, GLRaV-4/6, GLRaV-4/9, GRVFV, GRSPaV	12
		Cabernet Sauvignon SA125	GVA, GLRaV-3, GLRaV-4/9, GRSPaV	4
	SD	Shiraz BVRC12	GVA, GLRaV-3, GRSPaV	2
			GVA, GLRaV-3, GRVFV, GRSPaV	5
			GVA, GLRaV-4/9, GRVFV, GRSPaV	1

			GVA, GLRaV-3, GLRaV-4/9, GRVFV, GRSPaV	8
		Merlot	GVA, GLRaV-3, GRSPaV	1
Langhorne Creek	Asymptomatic	Shiraz	GRSPaV	14
			GRVFV, GRSPaV	1
	SD		GVA, GRSPaV	5
			GVA, GLRaV-4/9	7
			GVA, GLRaV-4/9, GRVFV, GRSPaV	2
			GVA, GLRaV-4/6, GRVFV, GRSPaV	1
Barossa	Mild LRD	heritage Shiraz	GVA*, GLRaV-1, GRSPaV	3
	Asymptomatic		GRSPaV	3
Coombe's	Mild LRD	Cabernet Sauvignon SA125	GVA, GLRaV-4/9, GRSPaV	2
	Asymptomatic	Shiraz BVRC12	GRVFV, GRSPaV	2

*Grapevine virus A detected in the grapevine with mild leafroll disease symptoms in Barossa valley.
#GRSPaV = grapevine rupestris stem pitting-associated virus, GRVFV = grapevine rupestris vein feathering virus, GLRaV-1, -3 = grapevine leafroll-associated virus 1, and 3, GLRaV-4/6, -4/9 = grapevine leafroll-associated virus 4 strains 6 and 9, GVA = grapevine virus A.

Table S4. Virus detection of samples tested by RT-PCR but not tested by high throughput sequencing, and the symptoms that were observed.

**Sample ID**[1]	**Virus status by RT-PCR**[2]	**Symptoms**[3]
WIL16	GRVFV, GRSPaV	Asymptomatic
WIL18	GLRaV-4/6, GRVFV, GRSPaV	Asymptomatic
WIL20	GLRaV-4/6, GRVFV, GRSPaV	Asymptomatic
WIL21	GLRaV-4/6, GRVFV, GRSPaV	Asymptomatic
WIL23	GLRaV-4/6, GRVFV, GRSPaV	Asymptomatic
WIL25	GLRaV-4/6, GRVFV, GRSPaV	Asymptomatic
WIL26	GRVFV, GRSPaV	Asymptomatic

WIL27	GLRaV-4/6, GRVFV, GRSPaV	Asymptomatic
WIL28	GLRaV-4/6, GRSPaV	Asymptomatic
WIL29	GLRaV-4/6, GLRaV-4/9, GRVFV, GRSPaV	Asymptomatic
WIL30	GLRaV-4/6, GLRaV-4/9, GRVFV, GRSPaV	Asymptomatic
WIL31	GVA#, GLRaV-4/9, GRVFV, GRSPaV	Asymptomatic
WIL32	GVA*#, GLRaV-4/9, GRVFV, GRSPaV	SD
WIL33	GVA#, GLRaV-3, GLRaV-4/9, GRVFV, GRSPaV	LRD
WIL34	GVA#, GLRaV-4/9, GRVFV, GRSPaV	Asymptomatic
WIL35	GVA#, GLRaV-3, GRVFV, GRSPaV	LRD
WIL36	GLRaV-3, GRVFV, GRSPaV	LRD
WIL37	GLRaV-3, GRVFV, GRSPaV	LRD
WIL38	GVA#, GLRaV-3, GLRaV-4/6, GLRaV-4/9, GRVFV, GRSPaV	LRD
WIL39	GVA#, GLRaV-4/6, GLRaV-4/9, GRVFV, GRSPaV	Asymptomatic
WIL40	GVA#, GLRaV-3, GLRaV-4/6, GLRaV-4/9, GRVFV, GRSPaV	LRD
WIL41	GLRaV-3, GLRaV-4/6, GLRaV-4/9, GRVFV, GRSPaV	LRD
WIL42	GLRaV-3, GLRaV-4/6, GRVFV, GRSPaV	LRD
WIL43	GVA#, GLRaV-3, GLRaV-4/6, GRVFV, GRSPaV	LRD
WIL44	GLRaV-4/9, GRVFV, GRSPaV	Asymptomatic
WIL45	GVA#, GLRaV-4/9, GRVFV, GRSPaV	Asymptomatic
WIL51	GVA*, GLRaV-3, GRVFV, GRSPaV	SD
WIL52	GVA*, GLRaV-3, GRVFV, GRSPaV	SD
WIL54	GLRaV-3, GLRaV-4/9, GRVFV, GRSPaV	LRD
WIL55	GLRaV-3, GLRaV-4/9, GRVFV, GRSPaV	LRD
WIL56	GVA#, GLRaV-4/9, GRVFV, GRSPaV	Asymptomatic
WIL57	GLRaV-4/9, GRSPaV	Asymptomatic
WIL58	GLRaV-4/9, GRSPaV	Asymptomatic
WIL59	GLRaV-4/9, GRVFV, GRSPaV	Asymptomatic

WIL60	GLRaV-3, GLRaV-4/9, GRVFV, GRSPaV	LRD
WIL61	GLRaV-3, GLRaV-4/9, GRVFV, GRSPaV	LRD
WIL62	GVA#, GLRaV-3, GLRaV-4/9, GRVFV, GRSPaV	LRD
WIL63	GLRaV-3, GLRaV-4/9, GRVFV, GRSPaV	LRD
WIL64	GLRaV-3, GLRaV-4/9, GRVFV, GRSPaV	LRD
WIL65	GLRaV-3, GRVFV, GRSPaV	LRD
WIL66	GLRaV-3, GLRaV-4/6, GLRaV-4/9, GRVFV, GRSPaV	LRD
WIL67	GVA#, GLRaV-3, GLRaV-4/6, GLRaV-4/9, GRVFV, GRSPaV	LRD
WIL68	GVA#, GLRaV-3, GLRaV-4/6, GLRaV-4/9, GRVFV, GRSPaV	LRD
WIL69	GLRaV-3, GLRaV-4/6, GLRaV-4/9, GRVFV, GRSPaV	LRD
WIL70	GLRaV-3, GLRaV-4/6, GLRaV-4/9, GRVFV, GRSPaV	LRD
WIL71	GLRaV-4/6, GLRaV-4/9, GRVFV, GRSPaV	Asymptomatic
WIL72	GLRaV-4/6, GLRaV-4/9, GRVFV, GRSPaV	Asymptomatic
WIL73	GLRaV-3, GLRaV-4/6, GRVFV, GRSPaV	LRD
WIL74	GLRaV-4/6, GRVFV, GRSPaV	Asymptomatic
WIL75	GLRaV-4/6, GRVFV, GRSPaV	Asymptomatic
WIL76	GLRaV-4/9, GRVFV, GRSPaV	Asymptomatic
WIL77	GLRaV-3, GRVFV, GRSPaV	LRD
WIL78	GLRaV-3, GLRaV-4/6, GRSPaV	LRD
WIL79	GLRaV-3, GRVFV, GRSPaV	LRD
WIL80	GLRaV-3, GRSPaV	LRD
WIL81	GLRaV-3, GRVFV, GRSPaV	LRD
LC1	GVA*, GLRaV-4/9, GRVFV, GRSPaV	SD
LC2	GVA*, GLRaV-4/9, GRSPaV	SD
LC3	GVA*, GLRaV-4/6, GRVFV, GRSPaV	SD
LC4	GVA*, GRSPaV	SD
LC8	GVA*, GLRaV-4/9, GRSPaV	SD

LC15	GVA*, GLRaV-4/9, GRVFV, GRSPaV	SD
LC17	GRSPaV	Asymptomatic
LC19 to LC30	GRSPaV	Asymptomatic
BV2	GVA*, GLRaV-1, GRSPaV	Mild LRD
BV4	GRSPaV	Asymptomatic
BV5	GRSPaV	Asymptomatic

[1] WIL = Willunga, LC = Langhorne Creek, BV = Barossa Valley. All grapevines listed are var. Shiraz

[2] GRSPaV = grapevine rupestris stem pitting-associated virus, GRVFV = grapevine rupestris vein feathering virus, GLRaV-1, -3 = grapevine leafroll-associated virus 1, and 3, GLRaV-4/6, -4/9 = grapevine leafroll-associated virus 4 strain 6 and 9, GVA = grapevine virus A.

[3] SD = Shiraz disease, LRD = leafroll disease (LRD)

*GVA positives by primer pairs Ah587/Ac995 and H7038/C7273.

GVA positives by primer pairs GVAgroupIIIF/R.

Table S5. Virus status detected by RT-PCR and metagenomic high-throughput sequencing (Meta-HTS) and the GVA phylogroup that was identified.

**Sample ID[1]**	**Symptoms[2]**	**Virus status by RT-PCR[4]**	**Virus status by Meta-HTS[4]**	**Phylo groups[3]**
WIL1	SD	GVA*#, GLRaV-3, GLRaV-4/9,GRVFV,GRSPaV	GVA, GLRaV-4/9, GRSPaV, GRVFV	II[3a] &III[3a]
WIL2	SD	GVA*#, GLRaV-3,GLRaV-4/9, GRVFV,GRSPaV	GVA, GLRaV-4/9, GRVFV, GRSPaV	II[3a] &III[3a]
WIL3	SD	GVA*#, GLRaV-3, GLRaV-4/9,GRVFV,GRSPaV	GVA, GLRaV-4/9, GRVFV, GRSPaV	II[3a] &III[3a]
WIL4	SD	GVA*, GLRaV-3, GLRaV-4/9,GRSPaV,GRVFV	GVA, GLRaV-3, GLRaV-4/9, GRVFV, GRSPaV	II[3a]
WIL5	SD	GVA*, GLRaV-3,GLRaV-4/9,GRVFV,GRSPaV	GVA, GLRaV-3, GLRaV-4/9, GRVFV, GRSPaV	II[3a]
WIL6	SD	GVA*, GLRaV-3,GLRaV-4/9,GRVFV,GRSPaV	GVA, GLRaV-3, GLRaV-4/9, GRVFV, GRSPaV	II[3a]
WIL7	SD	GVA*, GLRaV-3,GLRaV-4/9,GRVFV,GRSPaV	GVA, GLRaV-3, GLRaV-4/9, GRVFV, GRSPaV	II[3a]
WIL8	SD	GVA*#, GLRaV-3,GLRaV-4/9,GRSPaV,GRVFV	GVA, GLRaV-3, GLRaV-4/9, GRVFV, GRSPaV	II[3a] &III[3a]
WIL9	LRD	GVA#,GLRaV-3,GLRaV-4/9,GRVFV,GRSPaV	GVA, GLRaV-3, GLRaV-4/6, GLRaV-4/9, GRVFV, GRSPaV	III[3a]

WIL10	LRD	GVA#,GLRaV-3,GLRaV-4/6,GLRaV-4/9,GRVFV,GRSPaV	GVA, GLRaV-3, GLRaV-4/6, GLRaV-4/9,GRVFV,GRSPaV	III[3a]
WIL11	LRD	GVA#,GLRaV-3,GLRaV-4/6,GLRaV-4/9,GRVFV,GRSPaV	GVA, GLRaV-3, GLRaV-4/6, GLRaV-4/9, GRVFV,GRSPaV	III[3a]
WIL12	LRD	GVA#,GLRaV-3,GLRaV-4/6,GLRaV-4/9,GRVFV,GRSPaV	GVA, GLRaV-3, GLRaV-4/6, GLRaV-4/9,GRVFV,GRSPaV	III[3a]
WIL13	LRD	GLRaV-3, GLRaV-4/9,GRVFV,GRSPaV	GVA, GLRaV-3, GLRaV-4/5, GLRaV-4/9, GRVFV,GRSPaV	III[3a]
WIL14	LRD	GLRaV-3,GLRaV-4/6,GLRaV-4/9,GRVFV,GRSPaV	GLRaV-3, GLRaV-4/5, GLRaV-4/6, GLRaV-4/9, GRVFV,GRSPaV	Neg
WIL15	LRD	GLRaV-3, GLRaV-4/6,GRVFV,GRSPaV	GLRaV-3, GLRaV-4/5, GLRaV-4/6, GRVFV, GRSPaV	Neg
WIL17	Asymptomatic	GRVFV, GRSPaV	GRVFV, GRSPaV	Neg
WIL19	Asymptomatic	GLRaV-4/6,GRVFV,GRSPaV	GLRaV-4/6, GRVFV,GRSPaV	Neg
WIL22	Asymptomatic	GLRaV-4/6,GRVFV,GRSPaV	GLRaV-4/6, GRVFV,GRSPaV	Neg
WIL24	Asymptomatic	GRSPaV	GLRaV-4/6, GRVFV,GRSPaV	Neg
WIL47	SD	GVA*,GLRaV-3,GRVFV,GRSPaV	GVA, GLRaV-3, GRVFV, GRSPaV	III[3a]
WIL48	SD	GVA*,GLRaV-3,GRVFV,GRSPaV	GVA, GLRaV-3, GRSPaV	II[3a]
WIL49	SD	GVA*,GLRaV-3,GRVFV,GRSPaV	GVA, GLRaV-3, GRVFV, GRSPaV	II[3a]
WIL50	SD	GVA*,GLRaV-3, GRSPaV	GVA, GLRaV-3, GRSPaV	II[3b]
WIL53	SD	GVA*, GLRaV-3,GRSPaV	GVA, GLRaV-3, GRVFV, GRSPaV	II[3a]
LC5	SD	GVA*, GRSPaV	GVA, GLRaV-4/9, GRSPaV	II[3a]
LC6	SD	GVA*, GRSPaV	GVA, GLRaV-4/9, GRSPaV	II[3a]
LC7	SD	GVA*,GRSPaV	GVA, GLRaV-4/9, GRSPaV	II[3a]
LC9	SD	GVA*,GRSPaV	GVA, GLRaV-4/9, GRSPaV	II[3a]
LC10	SD	GVA*,GLRaV-4/9, GRSPaV,	GVA, GLRaV-4/9, GRSPaV	II[3a]

LC11	SD	GVA*,GLRaV-4/9, GRSPaV,	GVA, GLRaV-4/9, GRVFV, GRSPaV	II^{3a}
LC12	SD	GVA*,GLRaV-4/9, GRSPaV,	GVA, GLRaV-4/9, GRSPaV	II^{3b}
LC13	SD	GVA*,GLRaV-4/9, GRSPaV	GVA, GLRaV-4/9, GRSPaV	II^{3a}
LC14	SD	GVA*,GLRaV-4/9, GRSPaV	GVA, GLRaV-4/9, GRSPaV	II^{3a}
LC16	Asymptomatic	GRVFV, GRSPaV	GRVFV,GRSPaV	Neg
LC18	Asymptomatic	GRSPaV	GRVFV, GRSPaV	Neg
LC20	Asymptomatic	GRSPaV	GRSPaV	Neg
LC24	Asymptomatic	GRSPaV	GRSPaV	Neg
LC27	Asymptomatic	GRSPaV	GRVFV, GRSPaV	Neg
BV1	Mild LRD	GVA*, GLRaV-1, GRSPaV	GVA, GLRaV-1, GRVFV, GRSPaV	I^{3a}
BV3	Mild LRD	GVA*, GLRaV-1, GRSPaV	GVA, GLRaV-1, GRSPaV	I^{3b}
BV6	Asymptomatic	GRSPaV	GRVFV, GRSPaV	Neg
Cabw1	LRD	GVA*#, GLRaV-3, GLRaV-4/9, GRSPaV	GVA, GVF, GLRaV-3, GLRaV-4/9, GRSPaV, GRGV	II^{3b}&III^{3a}
Cabw2	LRD	GVA*#, GLRaV-3, GLRaV-4/9, GRSPaV	GVA, GVF, GLRaV-3, GLRaV-4/9, GRSPaV, GRGV	II^{3b}&III^{3b}
Cabw11	LRD	GVA*, GLRaV-3, GLRaV-4/9, GRSPaV	GVA, GLRaV-3, GLRaV-4/9, GRSPaV, GRVFV, GRGV	II^{3a}
Cabw12	LRD	GVA*#, GLRaV-3, GLRaV-4/9, GRSPaV	GVA, GLRaV-3, GLRaV-4/9, GRSPaV, GRVFV, GRGV	II^{3a}&III^{3b}
Melort1	SD	GVA#*, GLRaV-3, GRSPaV	GVA, GLRaV-3, GLRaV-4/9, GRSPaV	II^{3b}&III^{3b}
Shiraz_OR_P5	Asymptomatic	GRVFV, GRSPaV	GRVFV, GRSPaV	Neg

Shiraz_OR_P6	Asymptomatic	GRVFV, GRSPaV	GRVFV, GRSPaV	Neg
CabSA125_R3V30	LRD	GVA*#, GLRaV-4/9, GRSPaV,	GVA, GLRaV-4/9, GRSPaV, GRGV	II[3b]&III[3b]
CabSA125_R3V44	LRD	GVA*#, GLRaV-4/9, GRSPaV	GVA, GLRaV-4/9, GRSPaV	II[3b]&III[3b]

[1] WIL = Willunga, LC = Langhorne Creek, BV = Barossa Valley, CV= Coombe's Vineyard. All grapevines listed are var. Shiraz except Cabw = Cabernet Sauvignon from Willunga and CabSA125 = Cabernet Sauvignon clone SA125 from Coombe's Vineyard, Merlot = Merlot from Willunga.

[2] SD = Shiraz disease, LRD = leafroll disease (LRD)

[3] Phylogenetic groups were identified using contigs generated by Meta-HTS and phylogroups of each sample was identified based on the phylogroup of each GVA contig obtained from Meta-HTS (Tables S6 and S7). [3a] Phylogenetic group of GVA was identified using near complete sequences.

[3b] Phylogroup by partial sequences.

[4] GRSPaV = grapevine rupestris stem pitting-associated virus, GRVFV = grapevine rupestris vein feathering virus, GLRaV-1, -3 = grapevine leafroll-associated virus 1, and 3, GLRaV-4/5, -4/6, -4/9 = grapevine leafroll-associated virus 4 strains 5, 6 and 9, GVA = grapevine virus A, GVF = grapevine virus F, GRGV= grapevine red globe virus

*GVA positives by the generic assays using both primer pairs Ah587/Ac995 and H7038/C7273.

GVA positives by the group III specific assay using primer pairs GVAgroupIIIF/R.

Table S6. Basic statistics and list of contigs of the 50 grapevine samples sequenced by the NovaSeq (Illumina) instrument.

Sample ID[1]	Raw reads	Trimmed reads	Avg length after trim (bp)	Total no.contigs	Viroid contigs*	Virus contigs	Virus detected in this sample[4]	Exemplar isolate	Genus	Contig ID	Contig length (nts)	Average coverage (x)	Percentage of genome covered	Similarity to exemplar isolate
WIL1	8,844,230	8,819,752	130.4	87202	4	7	GVA[II]	Is151 (X75433)	*Vitivirus*	WIL1_N53_SD	7398	714.76	93.91%	5478/6903 (79.36%)
							GVA[III]	Is151 (X75433)	*Vitivirus*	WIL1_N54_SD	7348	460.279	23.11%	1366/1699 (80.4%)
							GLRaV-4/9	LR106 (FJ467503)	*Ampelovirus*	WIL1_N15	13954	1657.313	53.79%	5692/7439 (78.29%)
							GRSPaV	3138-07 (JX559646)	*Foveavirus*	WIL1_N23	8723	111.188	99.84%	8141/8712 (93.45%)

							GRSPaV	3138-07 (JX559646)	*Foveavirus*	WIL1_N35	8706	322.036	95.50%	6643/8333 (79.72%)
							GRVFV	Mauzac (KY513701)	*Marafivirus* (tentative)	WIL1_N74	6738	74.103	100.00%	5835/6744 (86.52%)
WIL2	7,711,315	7,692,915	130.7	82796	4	8	GVA[II]	Is151 (X75433)	*Vitivirus*	WIL2_N36_SD	7335	329.159	93.84%	5475/6898 (79.37%)
							GVA[III]	Is151 (X75433)	*Vitivirus*	WIL2_N40_SD	7183	545.375	20.91%	1207/1537 (78.53%)
							GLRaV-4/9	LR106 (FJ467503)	*Ampelovirus*	WIL2_N7	13837	1083.389	55.12%	5966/7623 (78.26%)
							GRSPaV	3138-07 (JX559646)	*Foveavirus*	WIL2_N21	8728	98.879	99.97%	8168/8723 (93.64%)
							GRSPaV	3138-07 (JX559646)	*Foveavirus*	WIL2_N22	8707	211.968	95.50%	6646/8333 (79.76%)
							GRVFV	Mauzac (KY513701)	*Marafivirus* (tentative)	WIL2_N88	5940	49.075	88.40%	5158/5949 (86.7%)
WIL3	7,515,198	7,487,210	131	80649	3	7	GVA[II]	Is151 (X75433)	*Vitivirus*	WIL3_N29_SD	7338	281.229	93.92%	5489/6904 (79.5%)
							GVA[III]	Is151 (X75433)	*Vitivirus*	WIL3_N27_SD	7366	1010.957	68.22%	3839/5015 (76.55%)
							GLRaV-4/9	LR106 (FJ467503)	*Ampelovirus*	WIL3_N6	13987	1825.827	55.14%	5973/7626 (78.32%)
							GRSPaV	3138-07 (JX559646)	*Foveavirus*	WIL3_N17	8712	125.018	99.85%	8142/8713 (93.45%)
							GRSPaV	3138-07 (JX559646)	*Foveavirus*	WIL3_N20	8286	168.353	90.60%	6263/7906 (79.22%)
							GRVFV	Mauzac (KY513701)	*Marafivirus* (tentative)	WIL3_N49	6718	86.218	100.00%	5745/6731 (85.35%)
							GRVFV	Mauzac (KY513701)	*Marafivirus* (tentative)	WIL3_N57	6383	87.836	95.02%	5559/6395 (86.93%)
WIL4	6,510,068	6,491,857	128.5	60366	2	18	GVA[II]	Is151 (X75433)	*Vitivirus*	WIL4_N28_SD	7322	513.125	93.95%	5481/6906 (79.37%)

							GLRaV-3	NY1 (AF037268)	*Ampelovirus*	WIL4_N4	18785	2069.145	100.00%	17548/17919 (97.93%)
							GLRaV-4/9	LR106 (FJ467503)	*Ampelovirus*	WIL4_N6	13984	1046.354	53.88%	5837/7451 (78.34%)
							GRSPaV	3138-07 (JX559646)	*Foveavirus*	WIL4_N17	8807	72.847	99.95%	8161/8722 (93.57%)
							GRSPaV	3138-07 (JX559646)	*Foveavirus*	WIL4_N18	8710	52.755	95.98%	6676/8375 (79.71%)
							GRVFV	Mauzac (KY513701)	*Marafivirus* (tentative)	WIL4_N417	3466	18.067	51.50%	2919/3466 (84.22%)
WIL5	5,471,483	5,450,378	124.9	59171	6	14	GVA[II]	Is151 (X75433)	*Vitivirus*	WIL5_N25_SD	7330	214.968	93.46%	5451/6870 (79.34%)
							GLRaV-3	NY1 (AF037268)	*Ampelovirus*	WIL5_N4	18634	647.413	100.00%	17549/17919 (97.94%)
							GLRaV-4/9	LR106 (FJ467503)	*Ampelovirus*	WIL5_N7	13900	471.646	53.88%	5838/7452 (78.34%)
							GRSPaV	3138-07 (JX559646)	*Foveavirus*	WIL5_N15	8753	138.559	95.98%	6678/8375 (79.74%)
							GRSPaV	3138-07 (JX559646)	*Foveavirus*	WIL5_N16	8710	102.693	99.77%	8149/8706 (93.6%)
							GRVFV	Mauzac (KY513701)	*Marafivirus* (tentative)	WIL5_N148	3874	24.803	57.64%	3316/3879 (85.49%)
WIL6	6,061,385	6,028,858	129	66387	2	11	GVA[II]	Is151 (X75433)	*Vitivirus*	WIL6_N29_SD	7356	428.922	93.61%	5464/6881 (79.41%)
							GLRaV-3	NY1 (AF037268)	*Ampelovirus*	WIL6_N4	18681	935.641	100.00%	17550/17919 (97.94%)
							GLRaV-4/9	LR106 (FJ467503)	*Ampelovirus*	WIL6_N6	13827	617.582	53.80%	5827/7440 (78.32%)
							GRSPaV	3138-07 (JX559646)	*Foveavirus*	WIL6_N14	8701	28.127	74.41%	5203/6493 (80.13%)
							GRSPaV	3138-07 (JX559646)	*Foveavirus*	WIL6_N21	8713	67.368	99.85%	8152/8713 (93.56%)

							GRVFV	Mauzac (KY513701)	*Marafivirus* (tentative)	WIL6_N5518	1262	9.194	18.72%	1054/1260 (83.65%)
WIL7	6,307,477	6,275,973	129.2	63855	4	14	GVA[II]	Is151 (X75433)	*Vitivirus*	WIL7_N29_SD	7336	148.576	93.97%	5505/6908 (79.69%)
							GLRaV-3	NY1 (AF037268)	*Ampelovirus*	WIL7_N5	18485	1159.452	99.92%	17531/17905 (97.91%)
							GLRaV-4/9	LR106 (FJ467503)	*Ampelovirus*	WIL7_N6	13857	780.61	53.80%	5823/7440 (78.27%)
							GRSPaV	3138-07 (JX559646)	*Foveavirus*	WIL7_N16	8761	104.034	92.36%	6463/8059 (80.2%)
							GRVFV	Mauzac (KY513701)	*Marafivirus* (tentative)	WIL7_N640	3082	22.426	45.66%	2625/3073 (85.42%)
WIL8	8,229,748	8,198,038	132.4	64064	2	17	GVA[II]	Is151 (X75433)	*Vitivirus*	WIL8_N27_SD	7361	302.566	92.57%	5395/6805 (79.28%)
							GVA[III]	Is151 (X75433)	*Vitivirus*	WIL8_N28_SD	7346	427.954	46.17%	2714/3394 (79.96%)
							GLRaV-3	NY1 (AF037268)	*Ampelovirus*	WIL8_N4	18569	1646.454	100.00%	17547/17919 (97.92%)
							GLRaV-4/9	LR106 (FJ467503)	*Ampelovirus*	WIL8_N7	13838	673.864	66.99%	7175/9265 (77.44%)
							GRSPaV	3138-07 (JX559646)	*Foveavirus*	WIL8_N16	8718	102.419	99.86%	8153/8714 (93.56%)
							GRSPaV	3138-07 (JX559646)	*Foveavirus*	WIL8_N17	8712	85.609	95.98%	6677/8375 (79.73%)
							GRVFV	Mauzac (KY513701)	*Marafivirus* (tentative)	WIL8_N376	3334	23.328	49.58%	2888/3337 (86.54%)
WIL9	7,436,140	7,409,318	131.8	75379	3	18	GVA[III]	Is151 (X75433)	*Vitivirus*	WIL9_N31_LRD	7364	386.726	23.10%	1357/1698 (79.92%)
							GLRaV-3	NY1 (AF037268)	*Ampelovirus*	WIL9_N3	18523	830.866	100.00%	17548/17919 (97.93%)
							GLRaV-4/6	LR106 (FJ467503)	*Ampelovirus*	WIL9_N25253	558	181.254	4.03%	431/558 (77.24%)

							GLRaV-4/9	LR106 (FJ467503)	*Ampelovirus*	WIL9_N5	13850	956.128	66.99%	7176/9265 (77.45%)
							GRSPaV	3138-07 (JX559646)	*Foveavirus*	WIL9_N19	8788	263.662	95.52%	6639/8335 (79.65%)
							GRSPaV	3138-07 (JX559646)	*Foveavirus*	WIL9_N21	8724	293.566	92.20%	6430/8045 (79.93%)
							GRSPaV	3138-07 (JX559646)	*Foveavirus*	WIL9_N22	8705	131.032	99.76%	8132/8705 (93.42%)
							GRVFV	Mauzac (KY513701)	*Marafivirus* (tentative)	WIL9_N4759	1464	33.023	21.72%	1257/1462 (85.98%)
WIL10	8,132,470	8,107,226	130.8	74459	3	11	GVA[III]	Is151 (X75433)	*Vitivirus*	WIL10_N41_LRD	7384	493.56	23.09%	1357/1697 (79.96%)
							GLRaV-3	NY1 (AF037268)	*Ampelovirus*	WIL10_N4	18319	830.274	99.91%	17531/17902 (97.93%)
							GLRaV-4/6	LR106 (FJ467503)	*Ampelovirus*	WIL10_N6	13957	803.428	55.67%	5938/7699 (77.13%)
							GLRaV-4/9	LR106 (FJ467503)	*Ampelovirus*	WIL10_N7	13854	577.642	66.99%	7177/9265 (77.46%)
							GRSPaV	3138-07 (JX559646)	*Foveavirus*	WIL10_N19	8788	186.984	99.97%	8169/8723 (93.65%)
							GRSPaV	3138-07 (JX559646)	*Foveavirus*	WIL10_N21	8706	162.929	95.91%	6668/8369 (79.67%)
							GRVFV	Mauzac (KY513701)	*Marafivirus* (tentative)	WIL10_N82	6175	43.267	90.85%	5119/6114 (83.73%)
WIL11	7,348,392	7,327,397	131.3	70213	3	18	GVA[III]	Is151 (X75433)	*Vitivirus*	WIL11_N43_LRD	7365	550.786	23.11%	1361/1699 (80.11%)
							GLRaV-3	NY1 (AF037268)	*Ampelovirus*	WIL11_N3	18692	843.165	100.00%	17546/17919 (97.92%)
							GLRaV-4/6	LR106 (FJ467503)	*Ampelovirus*	WIL11_N6	13925	898.052	55.65%	5934/7696 (77.1%)
							GLRaV-4/9	LR106 (FJ467503)	*Ampelovirus*	WIL11_N7	13815	521.005	53.65%	5804/7420 (78.22%)

							GRSPaV	3138-07 (JX559646)	*Foveavirus*	WIL11_N24	8710	154.401	95.48%	6640/8332 (79.69%)
							GRSPaV	3138-07 (JX559646)	*Foveavirus*	WIL11_N26	8459	208.263	89.26%	6209/7789 (79.71%)
							GRVFV	Mauzac (KY513701)	*Marafivirus* (tentative)	WIL11_N2122	2324	43.434	34.59%	2017/2328 (86.64%)
WIL12	7,188,416	7,170,862	130.9	70252	4	14	GVA[III]	Is151 (X75433)	*Vitivirus*	WIL12_N33_LRD	7343	302.104	23.29%	1363/1712 (79.61%)
							GLRaV-3	NY1 (AF037268)	*Ampelovirus*	WIL12_N3	18552	778.591	100.00%	17548/17919 (97.93%)
							GLRaV-4/6	LR106 (FJ467503)	*Ampelovirus*	WIL12_N5	13846	662.026	55.68%	5940/7700 (77.14%)
							GLRaV-4/9	LR106 (FJ467503)	*Ampelovirus*	WIL12_N6	13827	197.208	53.86%	5831/7449 (78.28%)
							GRSPaV	3138-07 (JX559646)	*Foveavirus*	WIL12_N21	8741	97.715	99.84%	8161/8712 (93.68%)
							GRSPaV	3138-07 (JX559646)	*Foveavirus*	WIL12_N22	8713	123.795	95.55%	6635/8338 (79.58%)
							GRVFV	Mauzac (KY513701)	*Marafivirus* (tentative)	WIL12_N352	3874	42.252	57.62%	3313/3878 (85.43%)
WIL13	6,529,218	6,499,025	114.9	50341	4	14	GVA[III]	Is151 (X75433)	*Vitivirus*	WIL13_N17_LRD	7380	386.722	23.11%	1365/1699 (80.34%)
							GLRaV-3	NY1 (AF037268)	*Ampelovirus*	WIL13_N3	18496	960.277	99.98%	17544/17916 (97.92%)
							GLRaV-4/5	LR106 (FJ467503)	*Ampelovirus*	WIL13_N7	13817	675.435	67.76%	7435/9371 (79.34%)
							GLRaV-4/9	LR106 (FJ467503)	*Ampelovirus*	WIL13_N6	13835	469.602	53.88%	5831/7451 (78.26%)
							GRSPaV	3138-07 (JX559646)	*Foveavirus*	WIL13_N13	8708	86.657	95.91%	6676/8369 (79.77%)
							GRSPaV	3138-07 (JX559646)	*Foveavirus*	WIL13_N14	8531	108.4	96.53%	7877/8423 (93.52%)

							GRVFV	Mauzac (KY513701)	*Marafivirus* (tentative)	WIL13_N74	4675	29.422	69.58%	3960/4683 (84.56%)
WIL14	7,491,817	7,475,282	131.3	74114	3	19	GLRaV-3	NY1 (AF037268)	*Ampelovirus*	WIL14_N4	18628	1148.426	100.00%	17551/17919 (97.95%)
							GLRaV-4/5	LR106 (FJ467503)	*Ampelovirus*	WIL14_N48	7283	172.25	16.98%	1994/2349 (84.89%)
							GLRaV-4/6	LR106 (FJ467503)	*Ampelovirus*	WIL14_N5	13892	599.815	57.92%	6166/8010 (76.98%)
							GLRaV-4/9	LR106 (FJ467503)	*Ampelovirus*	WIL14_N1317	2729	90.403	18.16%	1983/2512 (78.94%)
							GRSPaV	3138-07 (JX559646)	*Foveavirus*	WIL14_N19	8709	104.825	95.51%	6632/8334 (79.58%)
							GRSPaV	3138-07 (JX559646)	*Foveavirus*	WIL14_N20	8709	82.695	99.81%	8155/8709 (93.64%)
							GRSPaV	3138-07 (JX559646)	*Foveavirus*	WIL14_N21	8701	113.332	92.05%	6414/8032 (79.86%)
							GRVFV	Mauzac (KY513701)	*Marafivirus* (tentative)	WIL14_N338	4155	28.068	61.58%	3429/4144 (82.75%)
WIL15	7,789,197	7,711,086	126.7	63971	3	15	GLRaV-3	NY1 (AF037268)	*Ampelovirus*	WIL15_N4	18563	1298.621	100.00%	17549/17919 (97.94%)
							GLRaV-4/5	LR106 (FJ467503)	*Ampelovirus*	WIL15_N8	13095	179.913	67.61%	7415/9351 (79.3%)
							GLRaV-4/6	LR106 (FJ467503)	*Ampelovirus*	WIL15_N6	14017	678.683	57.92%	6166/8010 (76.98%)
							GRSPaV	3138-07 (JX559646)	*Foveavirus*	WIL15_N15	8740	102.612	99.84%	8158/8712 (93.64%)
							GRSPaV	3138-07 (JX559646)	*Foveavirus*	WIL15_N16	8796	249.931	93.15%	6508/8128 (80.07%)
							GRVFV	Mauzac (KY513701)	*Marafivirus* (tentative)	WIL15_N478	3358	41.478	49.91%	2853/3359 (84.94%)
WIL17	6,146,002	6,124,074	128.5	57552	3	13	GRSPaV	3138-07 (JX559646)	*Foveavirus*	WIL17_N13	8709	144.958	95.54%	6646/8337 (79.72%)

							GRSPaV	3138-07 (JX559646)	*Foveavirus*	WIL17_N14	8705	138.686	99.76%	8130/8705 (93.39%)
							GRVFV	Mauzac (KY513701)	*Marafivirus* (tentative)	WIL17_N147	4331	25.129	64.44%	3677/4337 (84.78%)
WIL19	7,469,836	7,439,964	129.8	60025	4	10	GLRaV-4/6	LR106 (FJ467503)	*Ampelovirus*	WIL19_N6	13930	546.193	56.25%	6011/7780 (77.26%)
							GRSPaV	3138-07 (JX559646)	*Foveavirus*	WIL19_N13	8763	92.843	99.84%	8163/8712 (93.7%)
							GRSPaV	3138-07 (JX559646)	*Foveavirus*	WIL19_N14	8709	163.499	95.54%	6645/8337 (79.7%)
							GRVFV	Mauzac (KY513701)	*Marafivirus* (tentative)	WIL19_N522	2942	23.692	43.76%	2505/2945 (85.06%)
WIL22	6,389,640	6,375,696	130.9	58491	4	15	GLRaV-4/6	LR106 (FJ467503)	*Ampelovirus*	WIL22_N5	13805	454.366	55.65%	5935/7696 (77.12%)
							GRSPaV	3138-07 (JX559646)	*Foveavirus*	WIL22_N12	8712	107.732	95.56%	6645/8339 (79.69%)
							GRSPaV	3138-07 (JX559646)	*Foveavirus*	WIL22_N13	8711	81.514	99.83%	8136/8711 (93.4%)
							GRVFV	Mauzac (KY513701)	*Marafivirus* (tentative)	WIL22_N148	4498	32.339	66.92%	3845/4504 (85.37%)
WIL24	7,389,330	7,361,985	133.5	68055	2	13	GLRaV-4/6	LR106 (FJ467503)	*Ampelovirus*	WIL24_N5	13867	642.107	55.68%	5939/7700 (77.13%)
							GRSPaV	3138-07 (JX559646)	*Foveavirus*	WIL24_N17	8740	78.037	95.56%	6664/8339 (79.91%)
							GRSPaV	3138-07 (JX559646)	*Foveavirus*	WIL24_N18	8702	51.304	99.74%	8122/8703 (93.32%)
							GRVFV	Mauzac (KY513701)	*Marafivirus* (tentative)	WIL24_N775	3070	17.366	45.29%	2585/3048 (84.81%)
LC5	3,641,648	3,577,621	125.2	39666	5	7	GVA[II]	Is151 (X75433)	*Vitivirus*	LC5_N12_SD	7339	136.552	92.49%	5401/6799 (79.44%)
							GLRaV-4/9	LR106 (FJ467503)	*Ampelovirus*	LC5_N5	13847	269.42	53.88%	5828/7452 (78.21%)

							GRSPaV	3138-07 (JX559646)	*Foveavirus*	LC5_N7	8704	70.512	99.75%	8161/8704 (93.76%)
LC6	5,893,931	5,869,862	127.4	57289	5	7	GVA[II]	Is151 (X75433)	*Vitivirus*	LC6_N19_SD	7348	137.147	93.61%	5456/6881 (79.29%)
							GLRaV-4/9	LR106 (FJ467503)	*Ampelovirus*	LC6_N6	13838	395.085	53.86%	5824/7449 (78.18%)
							GRSPaV	3138-07 (JX559646)	*Foveavirus*	LC6_N11	8712	84.278	99.84%	8167/8712 (93.74%)
							GRSPaV	3138-07 (JX559646)	*Foveavirus*	LC6_N12	8695	121.041	95.38%	6641/8323 (79.79%)
LC7	6,496,609	6,479,256	132.5	74472	5	7	GVA[II]	Is151 (X75433)	*Vitivirus*	LC7_N42_SD	7339	205.542	93.58%	5454/6879 (79.28%)
							GLRaV-4/9	LR106 (FJ467503)	*Ampelovirus*	LC7_N6	13886	429.195	53.85%	5823/7448 (78.18%)
							GRSPaV	3138-07 (JX559646)	*Foveavirus*	LC7_N24	8707	154.287	95.46%	6641/8330 (79.72%)
							GRSPaV	3138-07 (JX559646)	*Foveavirus*	LC7_N25	8700	79.634	92.05%	6426/8032 (80%)
LC9	7,067,771	7,040,778	116	54559	5	7	GVA[II]	Is151 (X75433)	*Vitivirus*	LC9_N19_SD	7336	107.851	93.93%	5464/6905 (79.13%)
							GLRaV-4/9	LR106 (FJ467503)	*Ampelovirus*	LC9_N5	13886	460.939	53.79%	5815/7439 (78.17%)
							GRSPaV	3138-07 (JX559646)	*Foveavirus*	LC9_N10	8746	49.552	99.85%	8166/8713 (93.72%)
							GRSPaV	3138-07 (JX559646)	*Foveavirus*	LC9_N13	8347	137.518	91.27%	6321/7964 (79.37%)
LC10	7,527,983	7,503,883	128.9	80310	4	6	GVA[II]	Is151 (X75433)	*Vitivirus*	LC10_N33_SD	7326	201.187	93.85%	5486/6899 (79.52%)
							GLRaV-4/9	LR106 (FJ467503)	*Ampelovirus*	LC10_N6	13855	721.857	53.88%	5846/7452 (78.45%)
							GRSPaV	3138-07 (JX559646)	*Foveavirus*	LC10_N15	8778	97.815	99.87%	8173/8715 (93.78%)

							GRSPaV	3138-07 (JX559646)	*Foveavirus*	LC10_N16	8755	207.959	95.73%	6673/8353 (79.89%)
							GRSPaV	3138-07 (JX559646)	*Foveavirus*	LC10_N17	8708	210.924	92.87%	6498/8104 (80.18%)
LC11	8,104,889	8,047,276	133.2	76962	3	8	GVA[II]	Is151 (X75433)	*Vitivirus*	LC11_N45_SD	7345	329.374	93.97%	5470/6908 (79.18%)
							GLRaV-4/9	LR106 (FJ467503)	*Ampelovirus*	LC11_N6	13853	1504.603	53.88%	5847/7451 (78.47%)
							GRSPaV	3138-07 (JX559646)	*Foveavirus*	LC11_N23	8824	208.7	95.32%	6643/8318 (79.86%)
							GRSPaV	3138-07 (JX559646)	*Foveavirus*	LC11_N24	8708	204.944	95.73%	6669/8353 (79.84%)
							GRSPaV	3138-07 (JX559646)	*Foveavirus*	LC11_N25	8699	122.517	99.69%	8144/8699 (93.62%)
							GRVFV	Mauzac (KY513701)	*Marafivirus* (tentative)	LC11_N65	6692	65.603	99.70%	5708/6710 (85.07%)
							GRVFV	Mauzac (KY513701)	*Marafivirus* (tentative)	LC11_N98	6116	70.257	90.82%	5118/6112 (83.74%)
LC12	5,786,465	5,765,655	130	60117	6	9	GVA[II]	Is151 (X75433)	*Vitivirus*	LC12_N124_SD	4608	128.498	62.78%	3681/4615 (79.76%)
							GLRaV-4/9	LR106 (FJ467503)	*Ampelovirus*	LC12_N5	13847	706.131	53.88%	5848/7452 (78.48%)
							GRSPaV	3138-07 (JX559646)	*Foveavirus*	LC12_N11	8763	60.616	99.85%	8172/8713 (93.79%)
							GRSPaV	3138-07 (JX559646)	*Foveavirus*	LC12_N12	8710	115.289	95.55%	6658/8338 (79.85%)
LC13	6,381,939	6,366,843	128.4	69862	5	7	GVA[II]	Is151 (X75433)	*Vitivirus*	LC13_N31_SD	7325	218.789	74.26%	4324/5459 (79.21%)
							GLRaV-4/9	LR106 (FJ467503)	*Ampelovirus*	LC13_N3	13836	599.363	53.62%	5792/7416 (78.1%)
							GRSPaV	3138-07 (JX559646)	*Foveavirus*	LC13_N16	8712	216.011	95.50%	6649/8333 (79.79%)

							GRSPaV	3138-07 (JX559646)	*Foveavirus*	LC13_N21	8176	210.55	21.12%	1443/1843 (78.3%)
LC14	7,454,524	7,438,758	133.3	80194	5	7	GVA[II]	Is151 (X75433)	*Vitivirus*	LC14_N49_SD	7361	461.658	94.08%	5474/6916 (79.15%)
							GVA[II]	Is151 (X75433)	*Vitivirus*	LC14_N51_SD	7305	465.173	93.55%	5444/6877 (79.16%)
							GLRaV-4/9	LR106 (FJ467503)	*Ampelovirus*	LC14_N6	13842	1053.333	53.88%	5826/7451 (78.19%)
							GRSPaV	3138-07 (JX559646)	*Foveavirus*	LC14_N30	8702	192.266	91.76%	6412/8007 (80.08%)
							GRSPaV	3138-07 (JX559646)	*Foveavirus*	LC14_N31	8702	119.366	99.72%	8154/8702 (93.7%)
							GRSPaV	3138-07 (JX559646)	*Foveavirus*	LC14_N33	8580	189.477	90.30%	6311/7880 (80.09%)
LC16	6,465,268	6,434,000	131.9	64080	12	7	GRSPaV	3138-07 (JX559646)	*Foveavirus*	LC16_N15	8739	195.301	99.97%	8169/8723 (93.65%)
							GRSPaV	3138-07 (JX559646)	*Foveavirus*	LC16_N16	8700	186.972	92.07%	6443/8034 (80.2%)
							GRVFV	Mauzac (KY513701)	*Marafivirus* (tentative)	LC16_N40	6476	79.407	96.51%	5431/6495 (83.62%)
LC18	7,790,861	7,770,023	132.5	69922	5	8	GRSPaV	3138-07 (JX559646)	*Foveavirus*	LC18_N16	8711	163.878	99.83%	8163/8711 (93.71%)
							GRSPaV	3138-07 (JX559646)	*Foveavirus*	LC18_N17	8709	330.907	95.48%	6636/8332 (79.64%)
							GRVFV	Mauzac (KY513701)	*Marafivirus* (tentative)	LC18_N18191	686	5.516	10.21%	571/687 (83.11%)
LC20	8,309,672	8,290,763	135.1	69527	3	6	GRSPaV	3138-07 (JX559646)	*Foveavirus*	LC20_N17	8779	252.426	99.97%	8179/8723 (93.76%)
							GRSPaV	3138-07 (JX559646)	*Foveavirus*	LC20_N22	8707	125.468	92.13%	6430/8039 (79.99%)
LC24	8,548,571	8,527,508	131.2	65729	3	5	GRSPaV	3138-07 (JX559646)	*Foveavirus*	LC24_N15	8727	244.805	99.84%	8159/8712 (93.65%)

							GRSPaV	3138-07 (JX559646)	*Foveavirus*	LC24_N19	8709	296.035	93.10%	6500/8124 (80.01%)
LC27	7,009,354	6,937,713	129.9	60293	3	8	GRSPaV	3138-07 (JX559646)	*Foveavirus*	LC27_N15	8704	142.126	99.75%	8163/8704 (93.78%)
							GRSPaV	3138-07 (JX559646)	*Foveavirus*	LC27_N16	8690	130.19	91.83%	6425/8013 (80.18%)
							GRVFV	Mauzac (KY513701)	*Marafivirus* (tentative)	LC27_N2994	1852	8.204	27.55%	1583/1854 (85.38%)
BV1	7,793,165	7,684,434	138.4	65632	5	14	GVA[I]	Is151 (X75433)	*Vitivirus*	BV1_N31_mild_LRD	7356	757.595	94.86%	5628/6973 (80.71%)
							GLRaV-1	1050 (JQ023131)	*Ampelovirus*	BV1_N3	18326	606.399	60.16%	9152/11226 (81.53%)
							GRSPaV	3138-07 (JX559646)	*Foveavirus*	BV1_N20	8709	426.948	95.55%	6663/8338 (79.91%)
							GRVFV	Mauzac (KY513701)	*Marafivirus* (tentative)	BV1_N2869	2158	10.479	32.08%	1850/2159 (85.69%)
BV3	6,823,261	6,801,634	130.8	45040	10	8	GVA[I]	Is151 (X75433)	*Vitivirus*	BV3_N260_mild_LRD	3217	333.538	43.98%	2678/3233 (82.83%)
							GLRaV-1	1050 (JQ023131)	*Ampelovirus*	BV3_N5	14324	181.667	49.67%	7681/9267 (82.89%)
							GRSPaV	3138-07 (JX559646)	*Foveavirus*	BV3_N9	8710	273.563	99.82%	8144/8710 (93.5%)
							GRSPaV	3138-07 (JX559646)	*Foveavirus*	BV3_N10	8657	450.294	94.90%	6600/8281 (79.7%)
BV6	6,021,139	5,998,506	131.4	47521	5	5	GRSPaV	3138-07 (JX559646)	*Foveavirus*	BV6_N11	8772	445.154	95.58%	6656/8340 (79.81%)
							GRVFV	Mauzac (KY513701)	*Marafivirus* (tentative)	BV6_N15461	597	4.702	8.87%	521/597 (87.27%)
Cabw1	7,450,095	7,434,263	129	74364	3	18	GVA[II]	Is151 (X75433)	*Vitivirus*	Cabw1_N153_LRD	5086	103.457	19.75%	1159/1452 (79.82%)
							GVA[III]	Is151 (X75433)	*Vitivirus*	Cabw1_N38_LRD	7343	379.453	23.28%	1374/1711 (80.3%)

							GVF	AUD46129 (JX105428)	*Vitivirus* (tentative)	Cabw1_N36	7520	79.977	99.55%	6734/7517 (89.49%)
							GLRaV-3	NY1 (AF037268)	*Ampelovirus*	Cabw1_N2	18502	1089.258	100.00%	17548/17919 (97.93%)
							GLRaV-4/9	LR106 (FJ467503)	*Ampelovirus*	Cabw1_N57	6596	197.098	46.91%	5106/6488 (78.7%)
							GRSPaV	3138-07 (JX559646)	*Foveavirus*	Cabw1_N45	6965	280.323	41.12%	2933/3588 (81.74%)
							GRGV	Graciano-T101 (KX171166)	*Maculavirus* (tentative)	Cabw1_N12057	919	6.351	13.64%	817/936 (87.29%)
Cabw2	5,679,378	5,670,120	118.4	60894	2	11	GVA^{II}	Is151 (X75433)	*Vitivirus*	Cabw2_N2273_LRD	1384	169.63	18.77%	1111/1380 (80.51%)
							GVA^{III}	Is151 (X75433)	*Vitivirus*	Cabw2_N6157_LRD	893	292.233	11.45%	716/842 (85.04%)
							GVF	AUD46129 (JX105428)	*Vitivirus* (tentative)	Cabw2_N17	7522	24.739	99.62%	6739/7522 (89.49%)
							GLRaV-3	NY1 (AF037268)	*Ampelovirus*	Cabw2_N1	18627	694.995	100.00%	17548/17919 (97.93%)
							GLRaV-4/9	LR106 (FJ467503)	*Ampelovirus*	Cabw2_N6	13818	174.28	53.86%	5832/7449 (78.29%)
							GRSPaV	3138-07 (JX559646)	*Foveavirus*	Cabw2_N11	8712	224.33	95.58%	6649/8340 (79.72%)
							GRSPaV	3138-07 (JX559646)	*Foveavirus*	Cabw2_N12	8712	139.557	99.84%	8151/8712 (93.56%)
							GRGV	Graciano-T101 (KX171166)	*Maculavirus* (tentative)	Cabw2_N17647	514	5.957	7.47%	439/513 (85.58%)
Cabw11	7,388,963	7,364,304	127.8	64873	3	15	GVA^{II}	Is151 (X75433)	*Vitivirus*	Cabw11_N36_LRD	7331	331.053	93.78%	5477/6894 (79.45%)
							GLRaV-3	NY1 (AF037268)	*Ampelovirus*	Cabw11_N3	18623	1219.735	100.00%	17543/17919 (97.9%)

							GLRaV-4/9	LR106 (FJ467503)	*Ampelovirus*	Cabw11_N31	13832	251.088	53.85%	5831/7448 (78.29%)
							GRSPaV	3138-07 (JX559646)	*Foveavirus*	Cabw11_N22	8738	227.72	99.95%	8169/8722 (93.66%)
							GRVFV	Mauzac (KY513701)	*Marafivirus* (tentative)	Cabw11_N1117	2784	16.009	41.38%	2384/2785 (85.6%)
							GRGV	Graciano-T101 (KX171166)	*Maculavirus* (tentative)	Cabw11_N40326	365	4.148	4.76%	277/327 (84.71%)
Cabw12	8,325,871	8,311,536	126.5	71483	3	16	GVA^{II}	Is151 (X75433)	*Vitivirus*	Cabw12_N47_LRD	7359	300.854	94.08%	5518/6916 (79.79%)
							GVA^{II}	Is151 (X75433)	*Vitivirus*	Cabw12_N52_LRD	7056	297.598	90.55%	5266/6656 (79.12%)
							GVA^{III}	Is151 (X75433)	*Vitivirus*	Cabw12_N650_LRD	3537	396.084	23.32%	1372/1714 (80.05%)
							GLRaV-3	NY1 (AF037268)	*Ampelovirus*	Cabw12_N4	18563	1096.171	100.00%	17548/17919 (97.93%)
							GLRaV-4/9	LR106 (FJ467503)	*Ampelovirus*	Cabw12_N7	13848	320.447	53.63%	5802/7417 (78.23%)
							GRSPaV	3138-07 (JX559646)	*Foveavirus*	Cabw12_N27	8752	273.132	99.89%	8155/8716 (93.56%)
							GRVFV	Mauzac (KY513701)	*Marafivirus (tentative)*	Cabw12_N1350	2775	17.089	41.32%	2377/2781 (85.47%)
							GRGV	Graciano-T101 (KX171166)	*Maculavirus (tentative)*	Cabw12_N10896	981	0	14.28%	877/980 (89.49%)
Merlot1	8,290,479	8,254,982	130.1	72852	2	15	GVA^{II}	Is151 (X75433)	*Vitivirus*	Merlot1_N121_SD	7356	175.8	93.50%	5434/6873 (79.06%)
							GVA^{III}	Is151 (X75433)	*Vitivirus*	Melort1_N1053_SD	2937	175.8	23.26%	1372/1710 (80.23%)
							GLRaV-3	NY1 (AF037268)	*Ampelovirus*	Merlot1_N5	18715	655.547	100.00%	17550/17919 (97.94%)

							GLRaV-4/9	LR106 (FJ467503)	*Ampelovirus*	Merlot1_N18	9339	271.871	51.33%	5526/7099 (77.84%)
							GRSPaV	3138-07 (JX559646)	*Foveavirus*	Merlot1_N23	8787	146.887	99.97%	8157/8723 (93.51%)
							GRSPaV	3138-07 (JX559646)	*Foveavirus*	Merlot1_N25	8708	244.398	95.53%	6655/8336 (79.83%)
WIL47	6,969,071	6,951,444	132.4	65411	4	7	GVA[III]	Is151 (X75433)	*Vitivirus*	WIL47_N43_SD	7352	138.221	23.29%	1371/1712 (80.08%)
							GLRaV-3	NY1 (AF037268)	*Ampelovirus*	WIL47_N4	18722	1722.427	100.00%	17548/17919 (97.93%)
							GRSPaV	3138-07 (JX559646)	*Foveavirus*	WIL47_N22	8726	369.074	95.65%	6662/8346 (79.82%)
							GRSPaV	3138-07 (JX559646)	*Foveavirus*	WIL47_N23	8710	215.087	99.82%	8142/8710 (93.48%)
							GRVFV	Mauzac (KY513701)	*Marafivirus* (tentative)	WIL47_N404	4019	10.897	59.69%	3427/4017 (85.31%)
WIL48	6,642,608	6,621,321	128.2	53212	5	4	GVA[II]	Is151 (X75433)	*Vitivirus*	WIL48_N20_SD	7322	116.535	93.78%	5487/6894 (79.59%)
							GLRaV-3	NY1 (AF037268)	*Ampelovirus*	WIL48_N4	18402	631.677	99.09%	17386/17756 (97.92%)
							GRSPaV	3138-07 (JX559646)	*Foveavirus*	WIL48_N11	8711	152.874	93.18%	6509/8131 (80.05%)
							GRSPaV	3138-07 (JX559646)	*Foveavirus*	WIL48_N12	8700	68.505	99.70%	8131/8700 (93.46%)
WIL49	8,918,793	8,887,686	132.7	76184	5	6	GVA[II]	Is151 (X75433)	*Vitivirus*	WIL49_N29_SD	7333	158.202	93.77%	5479/6893 (79.49%)
							GLRaV-3	NY1 (AF037268)	*Ampelovirus*	WIL49_N5	18622	730.866	99.94%	17539/17909 (97.93%)
							GRSPaV	3138-07 (JX559646)	*Foveavirus*	WIL49_N12	8712	299.75	95.45%	6656/8329 (79.91%)
							GRSPaV	3138-07 (JX559646)	*Foveavirus*	WIL49_N13	8712	172.718	99.84%	8139/8712 (93.42%)

							GRVFV	Mauzac (KY513701)	*Marafivirus* (tentative)	WIL49_N5272	1514	5.488	22.51%	1301/1515 (85.87%)
WIL50	4,070,795	4,045,084	127	28893	3	7	GVA^{II}	Is151 (X75433)	*Vitivirus*	WIL50_N27_SD	4284	34.486	19.59%	1156/1440 (80.28%)
							GRSPaV	3138-07 (JX559646)	*Foveavirus*	WIL50_N7	8706	127.646	95.50%	6657/8333 (79.89%)
WIL53	8,291,340	8,242,006	126.1	54153	3	8	GVA^{II}	Is151 (X75433)	*Vitivirus*	WIL53_N26_SD	7330	256.071	93.86%	5487/6900 (79.52%)
							GLRaV-3	NY1 (AF037268)	*Ampelovirus*	WIL53_N4	18561	1261.987	100.00%	17547/17919 (97.92%)
							GRSPaV	3138-07 (JX559646)	*Foveavirus*	WIL53_N15	8711	95.738	99.83%	8144/8711 (93.49%)
							GRSPaV	3138-07 (JX559646)	*Foveavirus*	WIL53_N16	8704	264.03	95.45%	6638/8329 (79.7%)
							GRVFV	Mauzac (KY513701)	*Marafivirus* (tentative)	WIL53_N433	3087	19.299	45.57%	2622/3067 (85.49%)
Shiraz_OR_P5	7,412,911	7378996	126.5	45848	2	8	GRSPaV	3138-07 (JX559646)	*Foveavirus*	Shiraz_OR_P5_N18	8722	1443.013	93.14%	6507/8127 (80.07%)
							GRSPaV	3138-07 (JX559646)	*Foveavirus*	Shiraz_OR_P5_N24	8715	890.954	99.87%	8156/8715 (93.59%)
							GRVFV	Mauzac (KY513701)	*Marafivirus* (tentative)	Shiraz_OR_P5_N304	4168	137.484	60.74%	3567/4088 (87.26%)
Shiraz_OR_P6	11,034,624	10968905	127.2	52144	3	4	GRSPaV	3138-07 (JX559646)	*Foveavirus*	Shiraz_OR_P6_N26	8802	682.444	99.97%	8162/8723 (93.57%)
							GRVFV	Mauzac (KY513701)	*Marafivirus* (tentative)	Shiraz_OR_P6_N564	3718	66.938	55.35%	3183/3725 (85.45%)
CabSA125_R3V30	7,734,941	7673531	126.9	41987	1	20	GVA^{II}	Is151 (X75433)	*Vitivirus*	CabSA125_3R30_N89_LRD	5731	222.553	71.64%	4126/5266 (78.35%)
							GVA^{III}	Is151 (X75433)	*Vitivirus*	CabSA125_3R30_N1944_LRD	2218	270.229	23.15%	1368/1702 (80.38%)
							GLRaV-4/9	LR106 (FJ467503)	*Ampelovirus*	CabSA125_3R30_N13	11333	452.9	53.14%	5745/7349 (78.17%)

							GRSPaV	3138-07 (JX559646)	*Foveavirus*	CabSA125_3R30_N21	8644	706.123	99.06%	8083/8644 (93.51%)
							GRGV	Graciano-T101 (KX171166)	*Maculavirus* (tentative)	CabSA125_3R30_N8761	1008	7.688	14.28%	877/980 (89.49%)
CabSA125_R3V44	6,838,643	6817153	128.4	49351	1	11	GVA[II]	Is151 (X75433)	*Vitivirus*	CabSA125_3R44_N55_LRD	6784	153.798	86.23%	5001/6339 (78.89%)
							GVA[III]	Is151 (X75433)	*Vitivirus*	CabSA125_3R44_N1182_LRD	2795	169.145	23.09%	1357/1697 (79.96%)
							GLRaV-4/9	LR106 (FJ467503)	*Ampelovirus*	CabSA125_3R44_N46	7274	218.01	51.89%	5602/7176 (78.07%)
							GRSPaV	3138-07 (JX559646)	*Foveavirus*	CabSA125_3R44_N24	8772	347.321	95.56%	6648/8339 (79.72%)
							GRSPaV	3138-07 (JX559646)	*Foveavirus*	CabSA125_3R44_N25	8710	142.797	99.82%	8125/8710 (93.28%)

[1] WIL = Willunga, LC = Langhorne Creek, BV = Barossa Valley, CV= Coombe's Vineyard. All grapevines listed are var. Shiraz except Cabw = Cabernet Sauvignon from Willunga and CabSA125 = Cabernet Sauvignon clone SA125 from Coombe's Vineyard, Merlot = Merlot from Willunga.
[2] Including number of contigs from grapevine yellow speckle viroid 1 and hop stunt viroid.
[3] Percentage of genome covered is calculated based on the pairwise alignment of this contig to the exemplar isolate of this virus using the "Blastn" command previously described.
[4] GRSPaV = grapevine rupestris stem pitting-associated virus, GRVFV = grapevine rupestris vein feathering virus, GLRaV-1, -3 = grapevine leafroll-associated virus 1, and 3, GLRaV-4/5, -4/6, -4/9 = grapevine leafroll-associated virus 4 strains 5, 6 and 9, GVA = grapevine virus A, GVF = grapevine virus F, GRGV= grapevine red globe virus

Table S7. Details of grapevine virus A (GVA) isolate sequences used for the phylogenetic analysis.

GenBank accession No (if available)	Isolate	Country	Variety or other host	Symptom[1]	RdRp[2] (ORF1)	MP[2] (ORF3)	CP[2] (ORF4)	RNA-binding[2] (ORF5)	Complete genome[2]
OP752632	BV1_N31_Mild_LRD	Australia	Shiraz	mild LRD	I	I	I	I&II	I
OP752610	Cabw12_N47_LRD	Australia	Cabernet Sauvignon	LRD	II	II	II	I&II	II

OP752625	Cabw12_N52_LRD	Australi a	Cabernet Sauvignon	LRD	II	II	II	N/A	II
OP752640	LC5_N12_SD	Australi a	Shiraz	SD	II	II	II	I&II	II
OP752637	LC6_N19_SD	Australi a	Shiraz	SD	II	II	II	I&II	II
OP752631	LC7_N42_SD	Australi a	Shiraz	SD	II	II	II	I&II	II
OP752611	LC9_N19_SD	Australi a	Shiraz	SD	II	II	II	I&II	II
OP752620	LC10_N33_SD	Australi a	Shiraz	SD	II	II	II	I&II	II
OP752618	LC11_N45_SD	Australi a	Shiraz	SD	II	II	II	I&II	II
OP752639	LC13_N31_SD	Australi a	Shiraz	SD	II	II	II	I&II	II
OP752616	LC14_N49_SD	Australi a	Shiraz	SD	II	II	II	I&II	II
OP752628	LC14_N51_SD	Australi a	Shiraz	SD	II	II	II	I&II	II
OP752613	WIL1_N53_SD	Australi a	Shiraz	SD	II	II	II	I&II	II
OP752635	WIL1_N54_SD	Australi a	Shiraz	SD	III	III	III	III	III
OP752633	WIL2_N36_SD	Australi a	Shiraz	SD	II	II	II	I&II	II
OP752608	WIL2_N40_SD	Australi a	Shiraz	SD	III	III	III	N/A	III
OP752609	WIL3_N27_SD	Australi a	Shiraz	SD	III	III	III	III	III
OP752623	WIL3_N29_SD	Australi a	Shiraz	SD	II	II	II	I&II	II

OP752626	WIL4_N28_SD	Australia	Shiraz	SD	II	II	II	I&II	II
OP752621	WIL5_N25_SD	Australia	Shiraz	SD	II	II	II	I&II	II
OP752642	WIL6_N29_SD	Australia	Shiraz	SD	II	II	II	I&II	II
OP752629	WIL7_N29_SD	Australia	Shiraz	SD	II	II	II	I&II	II
OP752615	WIL8_N27_SD	Australia	Shiraz	SD	II	II	II	I&II	II
OP752619	WIL8_N28_SD	Australia	Shiraz	SD	III	III	III	III	III
OP752622	WIL9_N31_LRD	Australia	Shiraz	LRD	III	III	III	III	III
OP752612	WIL10_N41_LRD	Australia	Shiraz	LRD	III	III	III	III	III
OP752641	WIL12_N33_LRD	Australia	Shiraz	LRD	III	III	III	III	III
OP752638	WIL13_N17_LRD	Australia	Shiraz	LRD	III	III	III	III	III
OP752624	WIL47_N43_SD	Australia	Shiraz	LRD	III	III	III	III	III
OP752617	WIL48_N20_SD	Australia	Shiraz	LRD	II	II	II	I&II	II
OP752636	WIL49_N29_SD	Australia	Shiraz	LRD	II	II	II	I&II	II
OP752627	WIL53_N26_SD	Australia	Shiraz	LRD	II	II	II	I&II	II
OP752614	Cabw11_N36_LRD	Australia	Cabernet Sauvignon	LRD	II	II	II	I&II	II
OP752634	Cabw1_N38_LRD	Australia	Cabernet Sauvignon	LRD	III	III	III	III	III

DQ855085	MSH18-1	SouthAfrica	Shiraz	SD-affected	N/A	N/A	I	I&II	N/A
KF013755	BVPN1-21c	USA	Pinot Noir	unknown	N/A	N/A	I	N/A	N/A
DQ855088	P163-1	SouthAfrica	Cinsaut Blanc	SD-negative	N/A	III	III	III	III
DQ787959	GTR1-1	SouthAfrica	Shiraz	SD-negative	III	III	III	III	III
DQ855086	GTR1-2	SouthAfrica	Shiraz	SD-negative	II	II	II	N/A	II
AF441235	JP98	SouthAfrica	Shiraz	SD-affected	N/A	N/A	II	I&II	N/A
MT070962	LC1-2	Australia	Shiraz	SD	II	II	II	N/A	II
MT070963	LC1-1	Australia	Shiraz	SD	II	II	II	II	II
MT070959	Malbec-Richter	Australia	Malbec	SD	N/A	N/A	II	N/A	N/A
DQ855087	BMo32-1	SouthAfrica	Merlot	SD-affected	II	II	II	I&II	II
DQ855083	KWVMo4-1	SouthAfrica	Merlot	SD-affected	II	II	II	I&II	II
DQ855082	P163-M5	SouthAfrica	Cinsaut Blanc	SD-affected	II	II	II	I&II	II
AY244516	pGR-5	SouthAfrica	*Nicotiana benthamiana*	SD-affected	I	I	I	N/A	I
AF007415	PA3	SouthAfrica	*N. benthamiana*	SD-affected	I	I	I	I&II	I

MT070960	BV1-2	Australia	Shiraz	SD-negative	N/A	N/A	I	N/A	N/A
MT070961	BV1-1	Australia	Shiraz	SD-negative	N/A	I	I	I&II	N/A
MG717802	Y170	Armenia	Ambarry	unknown	N/A	N/A	I	N/A	N/A
KF013847	PACF94-142-c07	USA	Cabernet Franc	unknown	N/A	N/A	I	N/A	N/A
JF754577	LN155	China	Golden finger	unknown	N/A	N/A	I	N/A	N/A
KF013785	H6TM2-3-c11	USA	Tamar S1	unknown	N/A	N/A	II	N/A	N/A
KF013833	LVCS92-06	USA	Cabernet Sauvignon	unknown	N/A	N/A	I	N/A	N/A
KF013764	CBPR116-c03	USA	Primitivo CL4	unknown	N/A	N/A	I	N/A	N/A
KF013840	LVMB92-10c11	USA	Malbec	unknown	N/A	N/A	I	N/A	N/A
EU008561	MT25-7	Czech	Muller Thurgau	unknown	N/A	I	I	N/A	N/A
EU008560	MT43-25	Czech	Muller Thurgau	unknown	N/A	N/A	I	N/A	N/A
MG717800	Y170	Armenia	Ambarry	unknown	N/A	N/A	I	N/A	N/A
KF594432	2	Macedonia	Vranec variety	unknown	N/A	N/A	I	I&II	N/A
KM233029	Rc3-6	Portugal	Ricoca	unknown	N/A	N/A	I	I&II	N/A
MK404721	TT2017-74-53	France	Pinot Noir	unknown	I	I	I	N/A	I

KM233033	S3-1	Portugal	Sousao	unknow n	N/A	N/A	I	I&II	N/A
JN860997	IH8	Poland	Vitis vinifera	unknow n	N/A	N/A	I	N/A	N/A
JN860998	IH11	Poland	Vitis sp.	unknow n	N/A	N/A	I	N/A	N/A
MK404720	TT2017-74-47	France	Pinot Noir	unknow n	I	I	I	N/A	I
KC962564	I327-5	SouthAf rica	Shiraz	SD-negativ e	I	I	I	I&II	I
KM233003	TM3-1	Portugal	Tinta Martins	unknow n	N/A	N/A	I	I&II	N/A
KM232996	BT3-11	Portugal	Bastardo Tinto	unknow n	N/A	N/A	I	N/A	N/A
KF013766	CBPR116-c12	USA	Primitivo CL4	unknow n	N/A	N/A	I	N/A	N/A
MF979533	VB-108	Croatia	Babica	unknow n	I	I	I	I&II	I
KX828703	TRAJ2-BR	Brazil	Trajadura	unknow n	N/A	I	I	I&II	I
DQ855081	GTR1SD-1	SouthAf rica	Shiraz	SD-affected	II	II	II	I&II	II
DQ855084	GTG11-1	SouthAf rica	Shiraz	SD-negativ e	I	I	I	I&II	I
X75433	Is-151	Italy	benthamiana	unknow n	I	I	I	I&II	I
MK404722	TT2017-79	France	Pinot Noir	unknow n	I	I	I	N/A	I
MG925333	P70	France	Pinot noir	unknow n	N/A	I	I	I&II	I

JX559641	3138-03	Canada	Vitis vinifera	unknown	III	III	III	III	III
LC617941	g13-C57*	Japan	Vitis sp.	unknown	III	III	III	I&II	III
LC617944	g13-C274	Japan	Vitis sp.	unknown	I	I	I	I&II	I
KF013813	LV94-02	USA	Vitis vinifera	unknown	N/A	N/A	I	N/A	N/A
MG977014	p26	Iran	Vitis vinifera	unknown	N/A	N/A	I	N/A	N/A
KF013799	SLWZF1-2c1	USA	Zinfandel	unknown	N/A	N/A	I	N/A	N/A
JF754576	LN141	China	Mars Seedless	unknown	N/A	N/A	I	N/A	N/A
AF441234	92-788	SouthAfrica	Cabernet Sauvignon	SD-affected	N/A	N/A	I	I&II	N/A
KM233013	MI3-15	Portugal	Malandra	unknown	N/A	N/A	I	I&II	N/A
KM233012	Ml3-12	Portugal	Malandra	unknown	N/A	N/A	I	I&II	N/A
KF594433	3	Macedonia	Frankovka	unknown	N/A	N/A	I	I&II	N/A
KF594434	4	Macedonia	Vranec	unknown	N/A	N/A	I	I&II	N/A
KF594435	5	Macedonia	Vranec	unknown	N/A	N/A	I	I&II	N/A
AB039841		Japan	Vitis sp.	unknown	N/A	N/A	I	N/A	N/A
AF441236	P163-1	SouthAfrica	Cinsaut Blanc	unknown	III	N/A	III	III	N/A
DQ911145	LQ58	China	Vitis sp	unknown	N/A	N/A	III	N/A	N/A

JF754566	LN9	China	Vitis vinifera	unknown	N/A	N/A	I	N/A	N/A
JF754567	LN37	China	Vitis vinifera	unknown	N/A	N/A	I	N/A	N/A
JF754568	LN43	China	Autumn black	unknown	N/A	N/A	I	N/A	N/A
JF754569	LN50	China	Vitis vinifera	unknown	N/A	N/A	I	N/A	N/A
JF754570	LN60	China	Rosario Bianco	unknown	N/A	N/A	I	N/A	N/A
JF754571	LN68	China	Yatomi Rosa	unknown	N/A	N/A	I	N/A	N/A
JF754572	LN107	China	Renitaka	unknown	N/A	N/A	I	N/A	N/A
JF754573	LN121	China	Vitis vinifera	unknown	N/A	N/A	I	N/A	N/A
JF754574	LN126	China	Vitis vinifera	unknown	N/A	N/A	I	N/A	N/A
JF754575	LN136	China	Vitis vinifera	unknown	N/A	N/A	I	N/A	N/A
JF754578	LN68-2	China	Yatomi Rosa	unknown	N/A	N/A	I	N/A	N/A
JN565033	IH10	Poland	Vitis vinifera	unknown	N/A	N/A	I	N/A	N/A
JN860999	IH23	Poland	Vitis vinifera	unknown	N/A	N/A	I	N/A	N/A
JN861000	IH27	Poland	Vitis vinifera	unknown	N/A	N/A	I	N/A	N/A
KF013756	BVPN1-2c	USA	Pinot Noir	unknown	N/A	N/A	I	N/A	N/A
KF013757	BVPN1-11G	USA	Pinot Noir	unknown	N/A	N/A	III	N/A	N/A

KF013758	BVPN2-8c	USA	Pinot Noir	unknown	N/A	N/A	III	N/A	N/A
KF013759	BVPN2-13c	USA	Pinot Noir	unknown	N/A	N/A	I	N/A	N/A
KF013760	BVPN2-b15E	USA	Pinot Noir	unknown	N/A	N/A	II	N/A	N/A
KF013761	BVPN2-b23E	USA	Pinot Noir	unknown	N/A	N/A	II	N/A	N/A
KF013762	BVPN2-b26B	USA	Pinot Noir	unknown	N/A	N/A	I	N/A	N/A
KF013763	CBPR116-2c	USA	Primitivo CL 4	unknown	N/A	N/A	III	N/A	N/A
KF013765	CBPR116-c09	USA	Primitivo CL 4	unknown	N/A	N/A	I	N/A	N/A
KF013767	CBSM119-2c	USA	Sami S1	unknown	N/A	N/A	I	N/A	N/A
KF013768	CBSM119-3c	USA	Sami S1	unknown	N/A	N/A	III	N/A	N/A
KF013770	CBSM119_c08	USA	Sami S2	unknown	N/A	N/A	I	N/A	N/A
KF013771	CSOT1_14c	USA	Cabernet Sauvignon	unknown	N/A	N/A	I	N/A	N/A
KF013772	CSOT1_4c	USA	Cabernet Sauvignon	unknown	N/A	N/A	II	N/A	N/A
KF013774	CSOT1-b50H	USA	Cabernet Sauvignon	unknown	N/A	N/A	III	N/A	N/A
KF013775	CSOT2-19c	USA	Cabernet Sauvignon	unknown	N/A	N/A	I	N/A	N/A
KF013776	CSOT2-2c	USA	Cabernet Sauvignon	unknown	N/A	N/A	III	N/A	N/A
KF013777	CSOT2-2G	USA	Cabernet Sauvignon	unknown	N/A	N/A	III	N/A	N/A

KF013779	H6TM2-3_2c2	USA	Tamar S1	unknow n	N/A	N/A	III	N/A	N/A
KF013780	H6TM2-3_3c1	USA	Tamar S1	unknow n	N/A	N/A	II	N/A	N/A
KF013781	H6TM2-3_3c2	USA	Tamar S1	unknow n	N/A	N/A	I	N/A	N/A
KF013782	H6TM2-3_c01	USA	Tamar S1	unknow n	N/A	N/A	II	N/A	N/A
KF013783	H6TM2-3_c07	USA	Tamar S1	unknow n	N/A	N/A	III	N/A	N/A
KF013784	H6TM2-3_c09	USA	Tamar S1	unknow n	N/A	N/A	III	N/A	N/A
KF013786	HHCS1_17c	USA	Cabernet Sauvignon	unknow n	N/A	N/A	III	N/A	N/A
KF013787	HHCS1_4c	USA	Cabernet Sauvignon	unknow n	N/A	N/A	II	N/A	N/A
KF013788	HHCS2_13c	USA	Cabernet Sauvignon	unknow n	N/A	N/A	III	N/A	N/A
KF013789	HHCS2_6c	USA	Cabernet Sauvignon	unknow n	N/A	N/A	II	N/A	N/A
KF013790	HHCS3_2c	USA	Cabernet Sauvignon	unknow n	N/A	N/A	I	N/A	N/A
KF013791	HHCS3_8c	USA	Cabernet Sauvignon	unknow n	N/A	N/A	III	N/A	N/A
KF013792	HHCS3_7c	USA	Cabernet Sauvignon	unknow n	N/A	N/A	II	N/A	N/A
KF013793	HHPN1_2c2	USA	Pinot Noir	unknow n	N/A	N/A	I	N/A	N/A
KF013794	HHPN1_9c	USA	Pinot Noir	unknow n	N/A	N/A	III	N/A	N/A
KF013795	HHPN1_16c	USA	Pinot Noir	unknow n	N/A	N/A	I	N/A	N/A

KF013796	HHPN1-115B	USA	Pinot Noir	unknow n	N/A	N/A	I	N/A	N/A
KF013797	HRPCH1_10c	USA	Chardonnay	unknow n	N/A	N/A	I	N/A	N/A
KF013798	SLWZF1-11c	USA	Zinfandel	unknow n	N/A	N/A	III	N/A	N/A
KF013800	SLWZF1-2c2	USA	Zinfandel	unknow n	N/A	N/A	II	N/A	N/A
KF013801	SLWZF1-5c2	USA	Zinfandel	unknow n	N/A	N/A	III	N/A	N/A
KF013802	SLWZF1-105E	USA	Zinfandel	unknow n	N/A	N/A	II	N/A	N/A
KF013803	SLWZF1-5D	USA	Zinfandel	unknow n	N/A	N/A	I	N/A	N/A
KF013804	SLWZF1-6	USA	Zinfandel	unknow n	N/A	N/A	II	N/A	N/A
KF013806	VHLM1_5c	USA	Lemberger	unknow n	N/A	N/A	I	N/A	N/A
KF013807	VHLM1_2c	USA	Lemberger	unknow n	N/A	N/A	I	N/A	N/A
KF013808	VHLM1-8D	USA	Lemberger	unknow n	N/A	N/A	I	N/A	N/A
KF013809	VHLM1-9D	USA	Lemberger	unknow n	N/A	N/A	I	N/A	N/A
KF013810	LREP100_9c	USA	Emperor	unknow n	N/A	N/A	III	N/A	N/A
KF013811	LREP100_c03	USA	Emperor	unknow n	N/A	N/A	III	N/A	N/A
KF013812	LV94-02_2c	USA	Vitis vinifera	unknow n	N/A	N/A	III	N/A	N/A
KF013814	LV94-02_c04	USA	Vitis vinifera	unknow n	N/A	N/A	I	N/A	N/A

KF013815	LV94-02_c09	USA	Vitis vinifera	unknown	N/A	N/A	I	N/A	N/A
KF013816	LVCH92-04_4c2	USA	Chardonnay	unknown	N/A	N/A	III	N/A	N/A
KF013817	LVCH92-04_7c	USA	Chardonnay	unknown	N/A	N/A	III	N/A	N/A
KF013818	LVCH92-04_c06	USA	Chardonnay	unknown	N/A	N/A	III	N/A	N/A
KF013819	LVCH92-07_3c	USA	Chardonnay	unknown	N/A	N/A	III	N/A	N/A
KF013820	LVCH92-07_2c1	USA	Chardonnay	unknown	N/A	N/A	III	N/A	N/A
KF013821	LVCH92-07_2c2	USA	Chardonnay	unknown	N/A	N/A	III	N/A	N/A
KF013822	LVCH92-07_c05	USA	Chardonnay	unknown	N/A	N/A	III	N/A	N/A
KF013823	LVCH92-07_c09	USA	Chardonnay	unknown	N/A	N/A	III	N/A	N/A
KF013824	LVCH92-09_2c1	USA	Chardonnay	unknown	N/A	N/A	III	N/A	N/A
KF013825	LVCH92-09_2c2	USA	Chardonnay	unknown	N/A	N/A	III	N/A	N/A
KF013826	LVCH92-09_3c	USA	Chardonnay	unknown	N/A	N/A	II	N/A	N/A
KF013827	LVCH92-09_c07	USA	Chardonnay	unknown	N/A	N/A	III	N/A	N/A
KF013828	LVCH94-04_10c	USA	Chardonnay	unknown	N/A	N/A	I	N/A	N/A
KF013829	LVCH94-04_c11	USA	Chardonnay	unknown	N/A	N/A	III	N/A	N/A
KF013830	LVCS92-06_2c1	USA	Chardonnay	unknown	N/A	N/A	I	N/A	N/A

KF013831	LVCS92-06_3c2	USA	Chardonnay	unknown	N/A	N/A	III	N/A	N/A
KF013832	LVCS92-06_2c2	USA	Chardonnay	unknown	N/A	N/A	II	N/A	N/A
KF013834	LVCS92-06_c04	USA	Chardonnay	unknown	N/A	N/A	III	N/A	N/A
KF013835	LVCS92-06_c08	USA	Chardonnay	unknown	N/A	N/A	III	N/A	N/A
KF013836	LVMB92-10_5c	USA	Malbec	unknown	N/A	N/A	I	N/A	N/A
KF013837	LVMB92-10_2c	USA	Malbec	unknown	N/A	N/A	III	N/A	N/A
KF013838	LVMB92-10_c05	USA	Malbec	unknown	N/A	N/A	I	N/A	N/A
KF013839	LVMB92-10_c08	USA	Malbec	unknown	N/A	N/A	I	N/A	N/A
KF013841	LVSB91-02_11c	USA	Malbec	unknown	N/A	N/A	I	N/A	N/A
KF013842	LVZT93-09_12c	USA	Zante	unknown	N/A	N/A	II	N/A	N/A
KF013843	PACF94-142_3c	USA	Cabernet Franc	unknown	N/A	N/A	I	N/A	N/A
KF013844	PACF94-142_4c	USA	Cabernet Franc	unknown	N/A	N/A	III	N/A	N/A
KF013845	PACF94-142_c02	USA	Cabernet Franc	unknown	N/A	N/A	III	N/A	N/A
KF013846	PACF94-142_c06	USA	Cabernet Franc	unknown	N/A	N/A	I	N/A	N/A
KF013848	PACF94-142_c08	USA	Cabernet Franc	unknown	N/A	N/A	I	N/A	N/A
KF013849	PACF94-142_c12	USA	Cabernet Franc	unknown	N/A	N/A	I	N/A	N/A

KF013850	PLCF95-413_4c	USA	Cabernet Franc	unknow n	N/A	N/A	III	N/A	N/A
KF013851	PLCF95-413_c02	USA	Cabernet Franc	unknow n	N/A	N/A	I	N/A	N/A
KF013852	PLCF95-413_c05	USA	Cabernet Franc	unknow n	N/A	N/A	III	N/A	N/A
KF013853	PLCF95-413_c06	USA	Cabernet Franc	unknow n	N/A	N/A	II	N/A	N/A
KF013854	PLCF95-413_c10	USA	Cabernet Franc	unknow n	N/A	N/A	II	N/A	N/A
LC387465	KGA	South Korea	Vitis sp.	unknow n	N/A	N/A	I	N/A	N/A
LK937667	K4	Greece	Aidani white	unknow n	N/A	N/A	III	N/A	N/A
LK937668	K12	Greece	Aidani red	unknow n	N/A	N/A	III	N/A	N/A
LK937669	T2-11	Greece	Koumariano	unknow n	N/A	N/A	III	N/A	N/A
LK937670	D9	Greece	Potamisi	unknow n	N/A	N/A	III	N/A	N/A
LK937671	P4-11	Greece	Aidani white	unknow n	N/A	N/A	I	N/A	N/A
LK937672	F15	Greece	Kakomauro	unknow n	N/A	N/A	III	N/A	N/A
LK937673	5Y	Greece	140 Ru	unknow n	N/A	N/A	I	N/A	N/A
LK937674	64	Greece	Vertzami	unknow n	N/A	N/A	I	N/A	N/A
LK937675	72-1	Greece	Cabernet franc	unknow n	N/A	N/A	III	N/A	N/A
LK937676	73	Greece	Roditis	unknow n	N/A	N/A	I	N/A	N/A

LK937677	75-1	Greece	Cabernet	unknown	N/A	N/A	I	N/A	N/A
LK937678	89-3	Greece	Attiki	unknown	N/A	N/A	I	N/A	N/A
LK937679	95-4	Greece	Roditis	unknown	N/A	N/A	I	N/A	N/A
LK937680	P14	Greece	Korithiaki stafida	unknown	N/A	N/A	I	N/A	N/A
LR794210	73a	Greece	Roditis	unknown	N/A	N/A	III	N/A	N/A
LR794211	K11	Greece	Mandilaria	unknown	N/A	N/A	III	N/A	N/A
LR794212	D24	Greece	Karabraimi	unknown	N/A	N/A	III	N/A	N/A
MG977013	p25	Iran	Vitis vinifera	unknown	N/A	N/A	I	N/A	N/A
KM232988	TL3-5	Portugal	Tinta Lameira	unknown	N/A	N/A	I	N/A	N/A
KM232989	TL3-7	Portugal	Tinta Lameira	unknown	N/A	N/A	I	N/A	N/A
KM232990	TL3-13	Portugal	Tinta Lameira	unknown	N/A	N/A	I	N/A	N/A
KM232991	TL3-31	Portugal	Tinta Lameira	unknown	N/A	N/A	I	N/A	N/A
KM233034	S3-4	Portugal	Sousao	unknown	N/A	N/A	I	I&II	N/A
KM233033	S3-1	Portugal	Sousao	unknown	N/A	N/A	I	I&II	N/A
KM233032	Rc3-1	Portugal	Ricoca	unknown	N/A	N/A	III	III	N/A
KM233031	Rc3-12	Portugal	Ricoca	unknown	N/A	N/A	I	I&II	N/A

KM233030	Rc3-7	Portugal	Ricoca	unknown	N/A	N/A	I	I&II	N/A
KM233029	Rc3-6	Portugal	Ricoca	unknown	N/A	N/A	I	I&II	N/A
KM233028	Rs3-16	Portugal	Roseira	unknown	N/A	N/A	I	I&II	N/A
KM233027	Rs3-15	Portugal	Roseira	unknown	N/A	N/A	III	III	N/A
KM233026	Rs3-10	Portugal	Roseira	unknown	N/A	N/A	III	III	N/A
KM233025	Rs3-7	Portugal	Roseira	unknown	N/A	N/A	I	I&II	N/A
KM233024	Rs3-6	Portugal	Roseira	unknown	N/A	N/A	I	I&II	N/A
KM233023	Rs3-5	Portugal	Roseira	unknown	N/A	N/A	I	I&II	N/A
KM233022	Rs3-3	Portugal	Roseira	unknown	N/A	N/A	I	I&II	N/A
KM233021	Rs3-2	Portugal	Roseira	unknown	N/A	N/A	I	I&II	N/A
KM233020	Rs3-1	Portugal	Roseira	unknown	N/A	N/A	I	I&II	N/A
KM233019	Ml3-3	Portugal	Roseira	unknown	N/A	N/A	III	III	N/A
KM232992	BT3-1	Portugal	Bastardo Tinto	unknown	N/A	N/A	I	N/A	N/A
KM232993	BT3-3	Portugal	Bastardo Tinto	unknown	N/A	N/A	I	N/A	N/A
KM232994	BT3-6	Portugal	Bastardo Tinto	unknown	N/A	N/A	I	N/A	N/A
KM232995	BT3-9	Portugal	Bastardo Tinto	unknown	N/A	N/A	I	N/A	N/A

KM232997	BT3-14	Portugal	Bastardo Tinto	unknow n	N/A	N/A	I	N/A	N/A
KM232998	BT3-33	Portugal	Bastardo Tinto	unknow n	N/A	N/A	I	N/A	N/A
KM232999	TF3-6	Portugal	Tinta Ferreira	unknow n	N/A	N/A	I	I&II	N/A
KM233000	TF3-3	Portugal	Tinta Ferreira	unknow n	N/A	N/A	III	III	N/A
KM233001	TF3-5	Portugal	Tinta Ferreira	unknow n	N/A	N/A	III	III	N/A
KM233002	TF3-7	Portugal	Tinta Ferreira	unknow n	N/A	N/A	III	III	N/A
KM233004	TM3-5	Portugal	Tinta Martins	unknow n	N/A	N/A	I	I&II	N/A
KM233005	TM3-6	Portugal	Tinta Martins	unknow n	N/A	N/A	I	I&II	N/A
KM233006	TM3-4	Portugal	Tinta Martins	unknow n	N/A	N/A	III	I&II	N/A
KM233007	Ml3-1	Portugal	Malandra	unknow n	N/A	N/A	I	I&II	N/A
KM233008	Ml3-4	Portugal	Malandra	unknow n	N/A	N/A	I	I&II	N/A
KM233009	Ml3-5	Portugal	Malandra	unknow n	N/A	N/A	I	I&II	N/A
KM233010	Ml3-10	Portugal	Malandra	unknow n	N/A	N/A	I	I&II	N/A
KM233011	Ml3-11	Portugal	Malandra	unknow n	N/A	N/A	I	I&II	N/A
KM233014	Ml3-16A	Portugal	Malandra	unknow n	N/A	N/A	I	I&II	N/A
KM233015	Ml3-9	Portugal	Malandra	unknow n	N/A	N/A	II	I&II	N/A

KM233016	Ml3-7	Portugal	Malandra	unknown	N/A	N/A	III	III	N/A
KM233017	Ml3-13	Portugal	Malandra	unknown	N/A	N/A	III	III	N/A
KM233018	Ml3-14	Portugal	Malandra	unknown	N/A	N/A	III	III	N/A
MK982553	M5v	South Africa	Cinsaut Blanc	SD-affected	II	II	II	I&II	II
MG717789	Y245-1	Turkey	Karasakis	unknown	N/A	N/A	I	N/A	N/A
MG717790	Y245-2	Turkey	Karasakis	unknown	N/A	N/A	I	N/A	N/A
MG717791	Y273-1	Turkey	Narince	unknown	N/A	N/A	I	N/A	N/A
MG717792	Y273-2	Turkey	Narince	unknown	N/A	N/A	I	N/A	N/A
MG717793	Y273-3	Turkey	Narince	unknown	N/A	N/A	I	N/A	N/A
MG717794	Y273-4	Turkey	Narince	unknown	N/A	N/A	I	N/A	N/A
MG717795	Y307-1	Ukraine	Tanagos Kosky	unknown	N/A	N/A	I	N/A	N/A
MG717796	Y307-2	Ukraine	Tanagos Kosky	unknown	N/A	N/A	I	N/A	N/A
MG717797	Y307-4	Ukraine	Tanagos Kosky	unknown	N/A	N/A	I	N/A	N/A
MG717798	Y161-1	Lebanon	Agmi Assouad	unknown	N/A	N/A	III	N/A	N/A
MG717799	Y161-2	Lebanon	Agmi Assouad	unknown	N/A	N/A	I	N/A	N/A
MG717801	Y170- 2	Armenia	Ambarry	unknown	N/A	N/A	I	N/A	N/A

MG717803	Y170- 4	Armenia	Ambarry	unknown	N/A	N/A	I	N/A	N/A
MG717804	Y170- 5	Armenia	Ambarry	unknown	N/A	N/A	I	N/A	N/A
MG717805	Y170- 6	Armenia	Ambarry	unknown	N/A	N/A	I	N/A	N/A
MG717806	Y172-1	Azerbaijan	Ara Hirna	unknown	N/A	N/A	I	N/A	N/A
MG717807	Y172-2	Azerbaijan	Ara Hirna	unknown	N/A	N/A	I	N/A	N/A
MG717808	Y172-3	Azerbaijan	Ara Hirna	unknown	N/A	N/A	I	N/A	N/A
MG717809	Y172-4	Azerbaijan	Ara Hirna	unknown	N/A	N/A	I	N/A	N/A
MG717810	95-177-1	Italy	Italia	unknown	N/A	N/A	I	N/A	N/A
MG717811	95-177-2	Italy	Italia	unknown	N/A	N/A	I	N/A	N/A
MG717812	95-177-3	Italy	Italia	unknown	N/A	N/A	I	N/A	N/A
MG717813	95-177-4	Italy	Italia	unknown	N/A	N/A	I	N/A	N/A
MG717814	Y199-1	USA	Black Monucca	unknown	N/A	N/A	III	N/A	N/A
MG717815	Y199-3	USA	Black Monucca	unknown	N/A	N/A	III	N/A	N/A
MG717816	Y199-4	USA	Black Monucca	unknown	N/A	N/A	III	N/A	N/A
MG717817	Y217-1	USA	Emperor	unknown	N/A	N/A	III	N/A	N/A
MG717818	Y217-2	USA	Emperor	unknown	N/A	N/A	III	N/A	N/A

MG717819	Y217-3	USA	Emperor	unknown	N/A	N/A	III	N/A	N/A
MG717820	Y217-4	USA	Emperor	unknown	N/A	N/A	III	N/A	N/A
MG717821	Y276-1	USA	Otscha Bala	unknown	N/A	N/A	I	N/A	N/A
MG717822	Y276-2	USA	Otscha Bala	unknown	N/A	N/A	I	N/A	N/A
MG717823	Y276-3	USA	Otscha Bala	unknown	N/A	N/A	I	N/A	N/A
MG717824	Y252-1	USA	Koudsi	unknown	N/A	N/A	III	N/A	N/A
MG717825	Y252-2	USA	Koudsi	unknown	N/A	N/A	III	N/A	N/A
MG717826	Y252-4	USA	Koudsi	unknown	N/A	N/A	III	N/A	N/A
MG717827	A94-1	USA	Servant	unknown	N/A	N/A	III	N/A	N/A
MG717828	A94-2	USA	Servant	unknown	N/A	N/A	II	N/A	N/A
MG717829	A94-4	USA	Servant	unknown	N/A	N/A	II	N/A	N/A
MG717831	SA125-2	Australia	Cabernet Sauvignon	unknown	N/A	N/A	II	N/A	N/A
MG717832	SA125-3	Australia	Cabernet Sauvignon	unknown	N/A	N/A	II	N/A	N/A
MG717833	Y258-1	Azerbaijan	Lilia Bidona	unknown	N/A	N/A	I	N/A	N/A
KF667501	IT-BA	Brazil	Italia	unknown	N/A	N/A	I	N/A	N/A
AY340581	SP	Brazil	Niagra rosada.	unknown	N/A	N/A	I	N/A	N/A

ON567250	A1892o	Russia	Vitis vinifera	unknown	I	I	I	I&II	I
MW309530	RSA-03-04_Agawam	South Africa	Agawam	unknown	I	I	I	I&II	I
MW309531	RSA-06-08_Blauer_Limberger	South Africa	Blauer Limberger	unknown	I	I	I	I&II	I
MW309532	RSA-07-15_Cataratto	South Africa	Cataratto	unknown	I	I	I	I&II	I
MW309533	RSA-09-06_Clairette_Egreneuse	South Africa	Clairette Egreneuse	unknown	N/A	III	III	III	III
MW309534	RSA-10-06_Cornifesto	South Africa	Cornifesto	unknown	I	I	I	I&II	I
MW309535	RSA-17-07_Kadarka	South Africa	Kadarka	unknown	I	I	I	I&II	I
MW309536	RSA-18-02_Limberger	South Africa	Limberger	unknown	I	I	I	I&II	I
MW309537	RSA-20-04_Mourvedre	South Africa	Mourvedre	unknown	III	III	III	III	III
MW309538	RSA-20-04_Mourvedre_2	South Africa	Mourvedre	unknown	I	I	I	I&II	I
MW309539	RSA-22-15_Mourisco_de_Semente	South Africa	Mourisco de Semente	unknown	I	I	I	I&II	I
MW309540	RSA-23-01_Muscat_d_Alexandrie_blanc	South Africa	Alexandrie blanc	unknown	I	I	I	I&II	I
MW309541	RSA-23-15_Muscat_St_Laurent	South Africa	Muscat Saint Laurent	unknown	III	III	III	III	III
MW309542	RSA-26-02_Petit_Sirah_Durif	South Africa	Petit Sirah	unknown	I	I	I	I&II	I
MW309543	RSA-33-06_Teoulier	South Africa	Teoulier	unknown	III	III	III	III	III

MW309544	strain RSA-33-06_Teoulier_2	South Africa	Teoulier	unknown	I	I	I	I&II	I
MW309545	RSA-33-07_Teroldego	South Africa	Teroldego	unknown	III	III	III	III	III
MW309547	RSA-45-07_Red_Globe_2	South Africa	Red Globe	unknown	I	I	I	I&II	I
MW309548	RSA-48-09_Tinta_Amarela	South Africa	Tinta Amarela	unknown	III	III	III	III	III
MW309549	RSA-48-09_Tinta_Amarela_2	South Africa	Tinta Amarela	unknown	I	I	I	I&II	I
MW309551	RSA-55-01_Staufer	South Africa	Staufer	unknown	II	II	II	I&II	II
MZ440717	12G456	Canada	Vitis vinifera	unknown	I	I	I	I&II	I
MZ440718	12G479A	Canada	Vitis vinifera	unknown	I	I	I	I&II	I
MZ440719	2G479B	Canada	Vitis vinifera	unknown	I	I	I	I&II	I
MZ440720	12G442	Canada	Vitis vinifera	unknown	III	III	III	III	III
MZ440721	12G446	Canada	Vitis vinifera	unknown	III	III	III	III	III
MZ440722	12G475	Canada	Vitis vinifera	unknown	I	I	I	I&II	I
NC_018458	AUD46129	USA	Vitis vinifera	N/A	N/A	N/A	N/A	N/A	N/A

[1] SD = Shiraz disease, LRD = leafroll disease (LRD), SD-affect and SD-negative isolates refer to when grapevines infected with these isolates, they show typical SD symptoms, or they remain asymptomatic [11,32].
[2] I = GVA^{I}, II = GVA^{II}, III = GVA^{III} , phylogroup of GVA by RdRp, MP, CP, RNA-binding genes and complete genome by phylogenetic analysis.
*Two isolates assigned to different phylogroup when using different gene regions of GVA.

Table S8. Details of the Australian grapevine leafroll associate virus 3 (GLRaV-3) isolates generated by metagenomic high-throughput sequencing and the publicly available isolates used for the phylogenetic analysis.

Phylogroup	% nt identities of CP gene of each phylogroup	Accession no.	Isolate	Country	Variety	%nt identity to NY1[1]
V	74.31-79.83	KY073323	8415A	Canada	Riesling	74.31%
		MH521097	Cha141	USA	Chardonnay	76.82%
		KM058745	GH24	South Africa	Cabernet Sauvignon	76.82%
		JX266782	3m-139	Australia	Sauvignon Blanc	76.86%
		KY707826	NdA121	Italy	Nero d'Avola	77.88%
		MK032068	Vdl	Canada	Vidal	77.92%
		MH521095	Cha138	USA	Chardonnay	78.66%
		MH521094	Cha137	USA	Chardonnay	78.66%
		KY764332	Trc139	USA	Chardonnay	78.66%
		JQ796828	3	USA	Merlot	78.87%
		JQ655296	GH30	South Africa	Cabernet Sauvignon	79.09%
		JQ655295	GH11	South Africa	Cabernet Sauvignon	79.41%
		MH521105	Gre233	Germany	Green Veltliner	79.72%
		MH521114	Mar239	USA	Marzemino	79.83%
IV	81.1-82.70	MH521098	Cha246	USA	Chardonnay	81.10%
		MH521091	Cab248	USA	Cabernet Sauvignon	81.21%
		MH521117	Pin244b	USA	Pinot Noir	81.21%
		KY073324	8415B	Canada	Riesling	81.42%

		MH796136	GLRaV3-ID45	USA	Cabernet Sauvignon	81.42%
		KY707824	Pro95	USA	11184	81.53%
		KY707825	Rod96	Greece	Roditis	81.53%
		KY764333	Trc138	USA	Chardonnay	81.63%
		MH521103	245	USA	Vitis vinifera	81.63%
		MH521109	Kat255b	Canada	Katelin	82.70%
III	91.4-91.72	KY886362	I-LR101	USA	Vitis vinifera	91.40%
		MF186605	I-LR101	USA	Vitis vinifera	91.40%
		GQ352633	PL-20	South Africa	Vitis sp.	91.51%
		MH521099	Cha246b	USA	Chardonnay	91.61%
		MH521100	Cha247	USA	Chardonnay	91.61%
		MH521118	Pin249	USA	Pinot noir	91.61%
		JQ423939	LN	China	Venus Seedless	91.72%
		MF991951	VB-108	Croatia	Babica	91.72%
		MH521104	Gor259	France	Goron de Bovernier	91.72%
II	92.46-92.78	MH814485	12G446	Canada	Vitis vinifera	92.46%
		EU259806	GP18	South Africa	Vitis sp.	92.46%
		MH814483	12G438A	Canada	Vitis vinifera	92.57%
		MH521115	Mer240	USA	Merlot	92.57%
		KY821093	AK6	Pakistan	Sugra one	92.57%
		MH814487	12G456	Canada	Vitis vinifera	92.68%
		KX701860	ISAB-BR	Brazil	Isabel	92.68%
		KJ174518		Israel	Vitis sp.	92.68%

		MH814486	12G448	Canada	Vitis vinifera	92.78%
		GQ352632	623	South Africa	Ruby Cabernet	92.78%
		KX756668	TC-BR	Brazil	Tardia de Caxias	92.78%
I	94.59-99.47	MH521102	185	USA	Vitis vinifera	94.59%
		MH814488	17VT16	Canada	Vitis vinifera	94.59%
		MK988555	Sau	China	Cabernet Sauvignon	97.77%
		EU344893	Cl-766	Chile	Merlot	99.04%
		MH521089	BHA172	Spain	BHAU 19-010	99.04%
		MH521088	AU173	Spain	AU 33-057	99.15%
		JX559645	3138-07	Canada	Vitis vinifera	99.15%
		MH796135	ID46	USA	Cabernet sauvignon	99.15%
		MH521110	Kis24	Hungary	Kishmish Vatkana	99.15%
		MN548393	SK933	Slovakia	Vitis vinifera	99.15%
		MK804765	NUB-BR	Brazil	Vitis sp. cv. BRS Nubia	99.15%
		KY821094	SL37-3	Pakistan	Italia	99.26%
		MH521090	Bla223	USA	Black Muscat Alexandria	99.26%
		GQ352631	621	South Africa	Cabernet Sauvignon	99.26%
		MH521101	Dk256	USA	DK03	99.26%
		OP752692	Cabw2_N1	Australia	Cabernet Sauvignon	99.26%
		OP752684	Cabw1_N2	Australia	Cabernet Sauvignon	99.26%
		MH814484	12G445	Canada	Vitis vinifera	99.26%
		MH814489	14G462	Canada	Vitis vinifera	99.26%
		MH521096	Cha138b	USA	Chardonnay	99.36%

KX756669	TRAJ-BR	Brazil	Trajadura	99.36%
MH521092	Cha103	USA	Chardonnay	99.36%
MH521111	Kis252	Hungary	Kishmish Vatkana	99.36%
MH521093	Cha106	USA	Chardonnay	99.36%
MH521119	Tou260	Portugal	Touriga Franca	99.36%
MH814491	14G466	Canada	Vitis vinifera	99.36%
MH521112	LN3204	USA	LN33	99.36%
MH521106	Ita218	USA	Italia	99.36%
MH521107	Ita219	USA	Italia	99.36%
OP752688	Merlot1_N5	Australia	Merlot	99.36%
OP752695	WIL47_N4	Australia	Shiraz	99.36%
OP752680	WIL53_N4	Australia	Shiraz	99.36%
OP752698	WIL48_N4	Australia	Shiraz	99.36%
OP752693	WIL49_N5	Australia	Shiraz	99.36%
OP752690	WIL12_N3	Australia	Shiraz	99.36%
OP752683	WIL5_N4	Australia	Shiraz	99.36%
OP752678	WIL6_N4	Australia	Shiraz	99.36%
OP752682	WIL7_N5	Australia	Shiraz	99.36%
OP752687	WIL4_N4	Australia	Shiraz	99.36%
OP752681	WIL8_N4	Australia	Shiraz	99.36%
OP752685	WIL10_N4	Australia	Shiraz	99.36%
OP752689	WIL9_N3	Australia	Shiraz	99.36%
OP752691	WIL50_N4	Australia	Shiraz	99.36%
OP752686	Cabw11_N3	Australia	Cabernet Sauvignon	99.36%
OP752696	WIL14_N4	Australia	Shiraz	99.36%

OP752677	WIL15_N4	Australia	Shiraz	99.36%
OP752679	Cabw12_N4	Australia	Cabernet Sauvignon	99.36%
MH814482	12G402	Canada	Vitis vinifera	99.47%
GU983863	WAMR	USA	Merlot	99.47%
MH521108	Kat255	Canada	Katelin	99.47%
MH521116	Pin244	USA	Pinot Noir	99.47%
MH814490	14G463	Canada	Vitis vinifera	99.47%
MH521113	Mal162	USA	Malbec	99.47%
OP752630	WIL11_N43	Australia	Shiraz	99.68%
JQ023131	GLRaV-1_1050	Canada	Vitis sp.	N/A

[1] Pairwise nucleotide identity of coat protein gene to the exemplar isolate NY1 (AF037268).

Table S9. Pairwise amino acid similarity of grapevine leafroll-associated virus 4 (GLRaV-4) to the exemplar isolate LR106 (GLRaV-4 strain 4, accession no. FJ467503).

GenBank accession No.	Isolate names	Strain of GLRaV-4	Country	Variety	% aa similarity of RdRp[1] to LR106	% aa similarity of HSP70h[1] to LR106	% aa similarity of CP[1] to LR106
KP313764	Ob	unclassified	Switzerland	Otcha bala	72.54	70.6	74.63
FJ907331	Carnelian		USA	Carnelian	N/A	66.73	77.94
AM182328	Pr	10	Greece	Mantilaria	81.47	N/A	N/A
KY940817	SW27-1		Pakistan	Tarakya	82.05	78.09	77.01
FJ467503	LR106	4	USA	Vitis sp.	100	100	100
KY821095	LH3		Pakistan	Autumn Royal	95.75	99.81	99.26

MF669483	WAMR-4		USA	Merlot	95.75	99.63	99.63
AY297819	-		USA	Vitis sp.	N/A	82.02	81.39
KJ810572	Man086		Spain	Mantua	86.87	82.02	81.75
MF669482	WALA-9		USA	Lagrein	87.26	82.21	82.48
OP752673	Cabw2_N6		Australia	Cabernet Sauvignon	87.04	81.65	82.12
OP752652	LC10_N6		Australia	Shiraz	87.04	81.65	82.12
OP752665	LC12_N5		Australia	Shiraz	87.04	81.65	81.75
OP752651	LC11_N6		Australia	Shiraz	87.04	81.65	81.75
OP752643	LC13_N3		Australia	Shiraz	87.23	81.27	82.48
OP752676	LC5_N5	9	Australia	Shiraz	87.23	81.27	82.48
OP752645	LC6_N6		Australia	Shiraz	87.23	81.27	82.48
OP752646	LC7_N6		Australia	Shiraz	87.23	81.27	82.48
OQ092720	LC1_N4		Australia	Shiraz	87.23	81.27	82.48
OP752675	LC9_N5		Australia	Shiraz	87.23	81.27	82.48
OP752670	LC14_N6		Australia	Shiraz	87.23	81.27	82.48
OP752660	WIL9_N5		Australia	Shiraz	86.85	81.65	81.39
OP752650	WIL10_N7		Australia	Shiraz	86.85	81.65	81.39

OP752666	WIL8_N7		Australia	Shiraz	86.85	81.65	81.39
OP752647	Cabw12_N7		Australia	Cabernet Sauvignon	87.04	81.46	82.12
OP752657	WIL12_N6		Australia	Shiraz	87.04	81.46	82.12
OQ092719	WIL11_N7		Australia	Shiraz	87.04	81.46	82.12
OP752659	Cabw11_N31		Australia	Cabernet Sauvignon	87.04	81.46	82.12
OP752653	WIL13_N6		Australia	Shiraz	87.04	81.46	82.12
OP752644	WIL7_N6		Australia	Shiraz	86.85	81.27	81.75
OP752668	WIL5_N7		Australia	Shiraz	87.23	81.46	82.12
OP752674	WIL6_N6		Australia	Shiraz	87.23	81.46	82.12
OP752648	WIL4_N6		Australia	Shiraz	87.23	81.46	82.12
OP752658	WIL1_N15		Australia	Shiraz	86.85	81.46	82.12
OP752669	WIL3_N6		Australia	Shiraz	87.04	81.46	82.48
OP752655	WIL2_N7		Australia	Shiraz	87.04	81.46	82.48
OP752654	WIL10_N6		Australia	Shiraz	85.49	81.09	80.29
OP752661	WIL12_N5	6	Australia	Shiraz	85.49	81.09	80.29
OP752672	WIL11_N6		Australia	Shiraz	85.3	81.09	80.29

OP752663	WIL19_N6		Australia	Shiraz	85.49	81.27	80.29
OP752649	WIL22_N5		Australia	Shiraz	85.3	81.27	80.29
OP752662	WIL24_N5		Australia	Shiraz	85.3	81.27	80.29
OP752664	WIL14_N5		Australia	Shiraz	85.49	81.27	80.66
OP752656	WIL15_N6		Australia	Shiraz	85.49	81.27	80.66
FJ467504	Estellat		USA	Vitis sp.	86.68	78.28	79.93
OP752671	WIL15_N8	5	Australia	Shiraz	86.85	81.27	82.12
OP752667	WIL13_N7		Australia	Shiraz	86.85	81.27	82.12
KY940818	LH3-1		Pakistan	Autumn Royal	85.91	82.02	81.75
FR822696	Y217		France	White Emperor	85.71	82.02	82.12
JX559639	3138-03		Canada	Vitis vinifera	85.71	82.21	82.12
KX828702	TRAJ1-BR		Brazil	Trajadura	86.68	82.02	83.21
MF669481	WASB-5		USA	Sauvignon Blanc	85.91	82.21	82.12
JX513893	1050-02		Canada	Vitis vinifera	85.91	82.4	81.75
KY940819	Bt26		Pakistan	Flame Seedless	86.49	82.02	81.02
AF414119	PMWaV-1	Out group	USA	unknown	N/A	N/A	N/A

[1] RdRp = RNA-dependent RNA polymerase (RdRp), HSP70h = heat shock protein 70 homologue, CP = coat protein
[2] Strains classified based on phylogenetic analysis of GLRaV-4 (Figure 5).

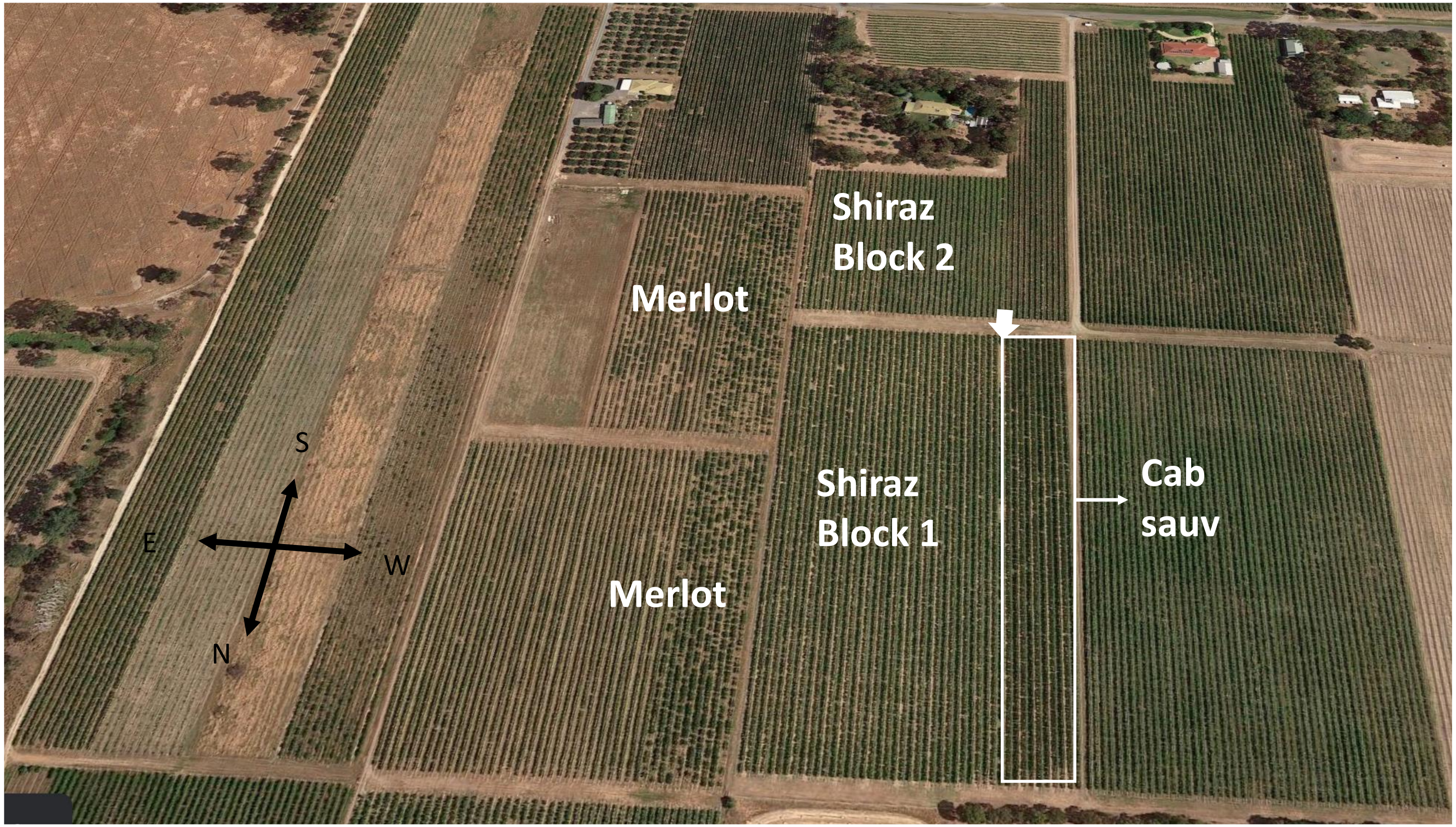

Figure S1. Vineyard map of the Willunga site. Shiraz grapevines from blocks 1 and 2 were used in the Metagenomic High Throughput Sequencing (Meta-HTS) andRT-PCR virus detection. The red arrow indicates Row 35 where symptoms of Shiraz disease (SD) were first discovered.

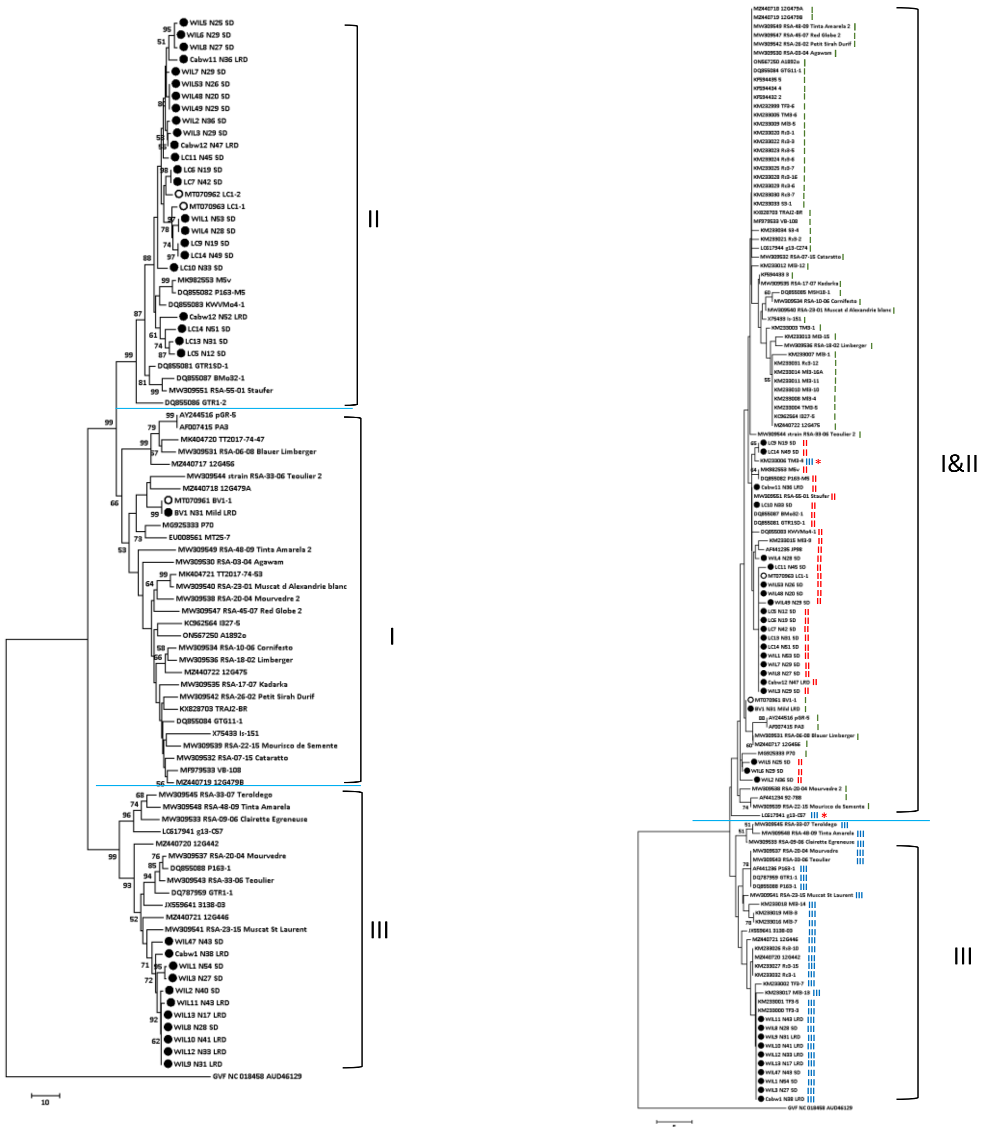
WIL5 N25 SD
WIL6 N29 SD
WIL8 N27 SD
Cabw11 N36 LRD
WIL7 N29 SD
WIL53 N26 SD
WIL48 N20 SD
WIL49 N29 SD
WIL2 N36 SD
WIL3 N29 SD
Cabw12 N47 LRD
LC11 N45 SD
LC6 N19 SD
LC7 N42 SD
MT070962 LC1-2
MT070963 LC1-1
WIL1 N53 SD
WIL4 N28 SD
LC9 N19 SD
LC14 N49 SD
LC10 N33 SD
MK982553 M5v
DQ855082 P163-M5
DQ855083 KWVMo4-1
Cabw12 N52 LRD
LC14 N51 SD
LC13 N31 SD
LC5 N12 SD
DQ855081 GTR1SD-1
DQ855087 BMo32-1
MW309551 RSA-55-01 Staufer
DQ855086 GTR1-2
II
AY244516 pGR-5
AF007415 PA3
MK404720 TT2017-74-47
MW309531 RSA-06-08 Blauer Limberger
MZ440717 12G456
MW309544 strain RSA-33-06 Teoulier 2
MZ440718 12G479A
MT070961 BV1-1
BV1 N31 Mild LRD
MG925333 P70
EU008561 MT25-7
MW309549 RSA-48-09 Tinta Amarela 2
MW309530 RSA-03-04 Agawam
MK404721 TT2017-74-53
MW309540 RSA-23-01 Muscat d Alexandrie blanc
MW309538 RSA-20-04 Mourvedre 2
MW309547 RSA-45-07 Red Globe 2
KC962564 I327-5
ON567250 A1892o
MW309534 RSA-10-06 Cornifesto
MW309536 RSA-18-02 Limberger
MZ440722 12G475
MW309535 RSA-17-07 Kadarka
MW309542 RSA-26-02 Petit Sirah Durif
KX828703 TRAJ2-BR
DQ855084 GTG11-1
X75433 Is-151
MW309539 RSA-22-15 Mourisco de Semente
MW309532 RSA-07-15 Cataratto
MF979533 VB-108
MZ440719 12G479B
I
MW309545 RSA-33-07 Teroldego
MW309548 RSA-48-09 Tinta Amarela
MW309533 RSA-09-06 Clairette Egreneuse
LC617941 g13-C57
MZ440720 12G442
MW309537 RSA-20-04 Mourvedre
DQ855088 P163-1
MW309543 RSA-33-06 Teoulier
DQ787959 GTR1-1
JX559641 3138-03
MZ440721 12G446
MW309541 RSA-23-15 Muscat St Laurent
WIL47 N43 SD
Cabw1 N38 LRD
WIL1 N54 SD
WIL3 N27 SD
WIL2 N40 SD
WIL11 N43 LRD
WIL13 N17 LRD
WIL8 N28 SD
WIL10 N41 LRD
WIL12 N33 LRD
WIL9 N31 LRD
III
GVF NC 018458 AUD46129
10
I&II
III

(a) (b)

Figure S2. Neighbor-joining phylogenetic analysis of amino acid (aa) sequences of full-length movement protein (MP) and RNA-binding (RB) genes of grapevine virus A (GVA) using metagenomic sequences and sequences from GenBank. (a) A neighbour-joining tree using full-length aa sequences of MP gene, (b) using complete aa sequences of CP gene. The phylogenetic trees were constructed using MEGA (7.0.26) software and the neighbour-joining method with 1000 bootstrap replicates. Only bootstrap values higher than 50% were shown. Sequences generated by the MiSeq and NovaSeq Meta-HTS experiments were labelled using circles and black dots, respectively. The variety, accession numbers and phylogroups assigned by each gene are listed in Table S6. Grapevine virus F (GVF) isolate AUD46129 (accession no. NC018458) was used as an outgroup. The phylogroup assigned by each gene was different for the two isolates (marked by *) in figure (b) and detailed in Table S6.

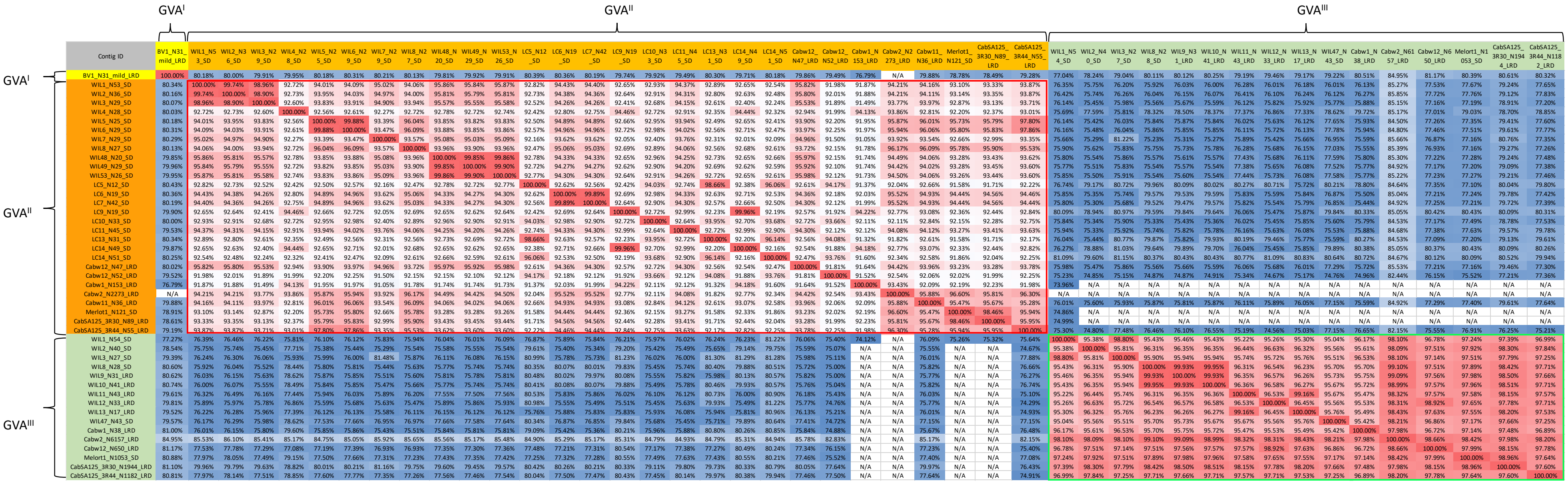

Figure S3. Color coded percentage of nucleotide (nt) pairwise identities of grapevine virus A (GVA) contigs in this study. GVA isolates from phylogenetic groups I, II and III are labeled as GVA^{I}, GVA^{II} and GVA^{III}. The contig IDs refer to Table S6. The pairwise identities were obtained by the the "blastn" function of Blast+. For contigs which do not share any comment genome region, identities are shown as N/A. The percentage of nt identities are color coded by the "3- color scale function" of Microsoft excel using blue (lowest value), white (midpoint) and red (highest value). The red and green squares show the sequence differences of GVA^{II} and GVA^{III} isolates between cv. Shiraz, Merlot and Cabernet Sauvignon, respectively.
